# Green Tea Leaves and Rosemary Extracts Selectively Induce Cell Death in Triple-Negative Breast Cancer Cells and Cancer Stem Cells and Enhance the Efficacy of Common Chemotherapeutics

**DOI:** 10.1155/2024/9458716

**Published:** 2024-01-25

**Authors:** Chris Raad, Abby Raad, Siyaram Pandey

**Affiliations:** Department of Chemistry and Biochemistry, University of Windsor, Windsor, Canada

## Abstract

While incredible medical advancements in chemotherapeutics development for cancer treatment have been made, the majority of these are not selective in their mechanism of action, leading to adverse effects. Given the systemic toxicity associated with these therapies, they are not well suited for long-term use. Natural health products, or NHPs, may provide a way to selectively target the oxidative and metabolic vulnerabilities in cancer cells. White tea (*Camelia sinensis*) and rosemary (*Salvia rosmarinus*) are two natural extracts that have been studied extensively for their medicinal properties. However, their anticancer activity and mechanism of action are yet to be fully elucidated. We have examined the extracts' cancer cell-killing ability as well as their interactions with common chemotherapeutics in MDA-MB-231 cells, a triple-negative breast cancer cell line, *in vitro*. Cell death measurement, morphological and biochemical characterization of apoptotic cell death, mechanisms of action (mitochondrial depolarization and oxidative stress), and immunofluorescence assays to estimate the percentage of cancer stem cells (CSCs) were performed following treatment with Synthite tea extract (STE) and rosemary extract (RE), provided by Synthite Industries Limited alone and in combination with cisplatin and paclitaxel. The key findings in this study are that STE and RE alone demonstrated very efficient anticancer activity against TNBC, and more importantly, the administration of the extracts in conjunction with cisplatin and paclitaxel sensitizes cancer cells to achieve enhanced cell death. In addition, CSCs were found to be sensitive to treatment with STE alone and in combination with RE and exhibited greater sensitivity to combination therapies compared to chemotherapeutic alone. The significance of these observations is that STE and RE, well-tolerated NHPs, have the potential to enhance the efficacy of current chemotherapeutics when combined, as well as prevent relapse for TNBC.

## 1. Introduction

Breast cancer is the most common cancer among women worldwide, making up a quarter of all cancer diagnoses [1]. Hormones play an important role in cancer's progression and, as such, present important targets for combatting the disease [2]. Breast cancer can generally be split into three subtypes: (i) receptor-positive tumours (estrogen and progesterone receptors), (ii) tumours with high levels of HER2 protein, and (iii) receptor-negative tumours with low HER2 production [2]. Breast cancers characterized by the lack of hormone receptors have a much poorer prognosis [2]. Belonging to the third subgroup, triple-negative breast cancer accounts for around one-tenth [3], or 10 to 15 percent, of all breast cancers [2]. It is named after its three deficiencies such as low HER2 production and negative estrogen and progesterone receptors [2]. The type of treatment administered is heavily influenced by hormone and protein production as they may be used as targets [2]. Lack of molecular targets makes TNBC an aggressive and challenging cancer to treat. Therefore, it is important to develop more effective treatment options [2].

While the advancements in medical technologies have resulted in improved healthcare outcomes for cancer patients, they face considerable limitations as the disease progresses [4]. With regard to preventative measures, breast cancer can be detected in its early stages by using screening methods [4]. At these stages, surgical methods may also be used to avoid further development [4]. Generally, tumourectomy is associated with high survival rates [5]. However, surgical methods risk aggravating the disease as the tumour can disseminate, advancing cancer to a late metastatic stage where surgical options are limited or futile [5]. Currently, chemotherapeutic regimens are the standard treatment plan once the disease has progressed to its later stages. Breast cancer is typically treated with cisplatin or paclitaxel, which are platinum-based and taxane-based drugs, respectively [6, 7]. While chemotherapeutics are an incredible medical advancement, they are not selective in their mechanism of action and may additionally target noncancerous cells [8, 9]. For example, paclitaxel is known to target the mitotic spindle assembly and cell division, which are the traits shared by both cancerous and healthy cells [8, 9].

Cancer stem cells (CSCs) are a self-renewing population of cells within a tumour [10–12]. CSCs have the potential to self-renew indefinitely due to the dysregulation of signalling pathways regulating self-renewal mechanisms [11]. In this manner, the proliferation of CSCs drives tumourigenesis or tumour formation [11]. Genotoxic agents such as cisplatin induce apoptosis in cancer cells by damaging DNA and triggering the DNA damage response [10]. CSCs have been found to promote DNA repair following DNA damage [13], thereby repopulating tumour masses after treatment with genotoxic chemotherapeutics [10]. CSCs have also been observed to exhibit chemotherapeutic resistance: differential survival of CSCs following treatment with chemotherapy has been found to increase the percentage of CSCs in progeny, subsequently leading to the formation of a chemoresistant tumour [14, 15]. As such, CSCs are thought to risk failure of anticancer chemotherapeutics and relapse in patients [10]. Current anticancer treatments target the entire population of cells within a tumour, but the proliferation potential of most tumour cells is limited [11]. Treatments that do not specifically target CSCs may shrink a tumour and later lead to tumour regrowth if a sufficient number of CSCs survive and proliferate indefinitely [11]. Therapies that specifically kill CSCs may deprive tumours of the ability to regenerate cells and grow [11, 16].

TNBCs seem enriched with CSCs compared to other breast cancer subtypes [17]. As chemotherapy resistance is frequently developed in TNBC patients [18] and around 40% of TNBC patients relapse after treatment with current chemotherapeutics [19], treatments targeting CSCs may improve TNBC patient's prognosis by attaining a stable tumour remission [10, 14].

CSCs are often identified by the presence of certain surface molecules [12, 20]. CSCs in TNBCs are associated with the phenotypes CD44+, CD24−, and ALDH1+ [21–23]. CD44+ and CD24− CSCs are thought to be highly invasive, contributing to tumourigenesis and metastasis [21, 23, 24]. In a study by Al-Hajj et al. [25], only 100 CSCs with the CD44+ and CD24− phenotype were needed to initiate tumour formation in mice, while tens of thousands of cells with other phenotypes were unable to form tumours. CD44 is a glycoprotein adhesion molecule [20] and is highly expressed in the MDA-MB-231 cell line [23, 24, 26]. This study will analyze the expression of the CD44 cell surface marker to investigate the ability of STE and RE, administered alone and in combination with current chemotherapeutics, to induce apoptosis in MDA-MB-231 CSCs.

The lack of selectivity displayed by chemotherapies may cause adverse side effects that ultimately lead to patient's death [27]. As such, a patient is limited to the number of cycles of chemotherapy they may take, as determined by the maximum lifetime dose [27]. In addition, many cancers are known to develop resistance to chemotherapeutic drugs, thus reducing their effectiveness [28]. Given the systemic toxicity and the risk of resistance associated with these treatments, they are not suitable as long-term treatments [27, 28].

Certain biochemical vulnerabilities can be exploited to target cancer cells selectively [29, 30]. Due to their hyperactive metabolic activity, cancer cells generally display increased intracellular levels of reactive oxygen species (ROS) [29]. ROS are highly reactive, unstable oxygen-containing molecules that may interact with other molecules in the cells [29]. While their occurrence is important for healthy cellular processes, an excess of ROS may damage the DNA, RNA, and proteins [29]. Naturally, there are protective cellular mechanisms tasked with managing the balance of ROS [29, 30]. However, cells will undergo apoptosis once the levels of oxidative stress are pushed beyond the level by which the body may cope [30]. Previous works have shown that various NHPs can exploit these oxidative weaknesses to selectively induce apoptosis without harming normal cells [31]. Furthermore, the differences between cancerous and noncancerous cell mitochondrial activity may also be exploited [30]. The Warburg effect explains that cancerous cells prefer metabolism via glycolysis which leads to a buildup of lactic acid within the cytosol [32]. An acidic cytosol is one of the multiple factors leading to the hyperpolarization of cancerous mitochondria [33]. Therapeutics that target the mitochondrial membrane potential (MMP) do so by initiating an internal stress stimulus which causes proapoptotic proteins to translocate to and permeabilize the outer mitochondrial membrane [34]. Once permeabilized, the mitochondria become depolarized and apoptogenic factors from the intermembrane space are released into the cytosol of the cell, ultimately leading to apoptosis [34].

Natural health products (NHPs) or botanical substances have served older human civilizations for a wide range of medicinal purposes [35]. Over the course of hundreds of years, the ancient Chinese, Ayurvedic, and First Nation tribes used plant-based medicines to treat acute and chronic diseases [35, 36]. Their long history of medicinal use has warranted further investigation by researchers across the world [36]. For example, turmeric, a plant native to southeastern Asia, has been extensively shown to contain phytochemicals with antioxidant, anti-inflammatory, antimutagenic, antimicrobial, and anticancer properties. Pertaining to the oncological field, isolated compounds from NHPs account for three-quarters of current chemotherapeutic drugs [31].

As their mechanisms have not fully been elucidated, medical professionals are rightfully skeptical of their efficacy in a clinical setting [35, 36]. The skepticism surrounding NHPs is further amplified by the lack of information on their interactions with common chemotherapeutic poses [31]. Along with their therapeutic value, NHPs are known to be safe for consumption in humans with minimal risk and thus are a promising candidate for further research on their anticancer activities [31].

*Camellia sinensis*, a plant native to China, belongs to the tea plant family Theaceae [37]. The leaves of *C. sinensis* are used to prepare a variety of teas, for example, green, black, oolong, and white tea [37]. The plant contains a multitude of bioactive compounds such as polyphenols, flavonoids, and vitamins [37]. As such, these teas have been linked with therapeutic properties against cardiovascular diseases, cancers, and inflammatory diseases [37]. Previous studies have demonstrated the anticancer activity of *Camellia sinensis* [38]. The extract has been shown to selectively target MDA-MB-231 cells, thereby reducing cell viability in the TNBC cells while having no toxic effect on noncancerous cells [38]. The extract was also found to reduce cell migration in MDA-MB-231 by 50%, which has implications for addressing tumour progression [38].

Synthite tea extract (STE) is an extract prepared from fresh leaves of *Camellia sinensis* by using a proprietary procedure described by Synthite Industries Ltd. The minimalistic preparation results in a high retention of polyphenol antioxidants such as epicatechins [39]. These phytochemicals are known to interact with and quench ROS *in vitro* [39]. As explained in an earlier section, ROS is involved in the pathology of several diseases [29], as such the epicatechins in STE may have therapeutic value [39]. Specifically, epigallocatechin-3-gallate has been shown to have apoptosis-inducing and antiproliferative activity against different cancers [40].

*Salvia rosmarinus*, or more commonly known as rosemary, is a shrub-like plant that is native to the Mediterranean region [41]. The plant is characterized by its evergreen and needle-shaped leaves, and it belongs to the mint family *Lamiaceae* [41]. Aside from being a commonly used spice, the plant has long been used in medicinal settings due to its anti-inflammatory, antioxidant, antimicrobial, antiproliferative, and antitumour activities [41]. Among other bioactive compounds, RE contains phenolic diterpenes such as carnosol, carnosic acid, and rosmarinic acid [42].

Rosemary has been proven to display powerful anticancer efficacy in numerous cancers *in vitro*, such as cancer of the lung, prostate, liver, and breast [42]. Carnosic acid appears to be the plant's primary anticancer component, as it was found to exhibit antiproliferative effects in cancer cells [41]. As well, rosmarinic acid had a significant anticancer activity against several cell lines [42]. In MDA-MB-231 cells, the extract was found to exhibit an antiproliferative effect by inhibiting cell growth in a dose-dependent manner [43]. Previous studies have also shown that the extract exhibits an antimigratory effect on MDA-MB-231 cells by reducing cell motility [43]. Previous studies thus demonstrate rosemary extract's potential as an anticancer treatment [43], and so its mechanisms of action as well as its interactions with common chemotherapeutics should be investigated.

TNBCs were chosen for this study due to their difficulty to treat and aggressivity, which creates a need to develop safer and more effective therapy. Compounds from *Camellia sinensis* and *Salvia rosmarinus* have been shown to exhibit anticancer activity in the past [38, 43]. The advantage of using the total extract is that they are well-tolerated, while purified compounds may be toxic. Furthermore, total extracts contain multiple compounds which may have synergistic effects on anticancer activity [36]. We have previously shown that STE was effective in inducing apoptosis in lymphoma cells [44]. However, the efficacy of STE and RE alone has not been studied in detail in MDA-MB-231 cells. Furthermore, STE and RE may sensitize TNBC cells to chemotherapeutics, leading to a more effective treatment of this aggressive cancer. Patients are also limited in the number of chemotherapeutic cycles they may undergo [27], while STE and RE are well-tolerated and can be administered long-term. This study aims to investigate the anticancer effects of STE and RE in MDA-MB-231, a TNBC cell line, as well as their interactions with common chemotherapeutics. We hypothesize that the multiple phytochemicals in these well-tolerated extracts have their own anticancer effects and enhance the efficacy of common chemotherapeutics if administered together.

## 2. Materials and Methods

### 2.1. Synthite Tea and Rosemary Extraction and Preparation

Both extracts STE and RE used in this project are extracted and prepared by Synthite Industries Private Ltd., a privately held company based in Kochi, India. Synthite obtains these materials from its own tea and rosemary forms and produces these extracts on a large scale. Details pertaining to the extraction and preparation of STE and RE are owned by Synthite Industries and are not publicly disclosable. Synthite has provided HPLC analysis profiles and certificate of analysis (COA) for both extracts. This information is provided in Supplemental files. These extracts have been approved for human consumption in India as supplements.

A fine powder of Synthite tea was dissolved in distilled water to make an aqueous extract. Rosemary, in a CO_2_ supercritical fluid form, was dissolved in DMSO.

### 2.2. Cell Culture and Treatment

The breast cancer cell line MDA-MB-231 (ATCC® HTB-26™) was cultured in Eagle's Minimum Essential Medium (EMEM) (ATCC® 30–2003™) supplemented with 10% (v/v) fetal bovine serum (FBS) and 0.4% (v/v) gentamicin. The normal colon mucosa cell line (ATCC® CRL-1831™) was cultured in Medium M3 Base (INCELL Corp. M300F-500) supplemented with 10% (v/v) fetal bovine serum (FBS) and 0.4% (v/v) gentamicin. These cells were grown and maintained in an incubator, set at 37°C, with an atmosphere containing 5% CO_2_ and 95% humidity.

The normal colon mucosa cell line NCM-460 (ATCC® CRL-1831™) was cultured in Medium M3 Base (INCELL Corp. M300F-500) supplemented with 10% (v/v) fetal bovine serum (FBS) and 0.4% (v/v) gentamicin. These cells were grown and maintained in an incubator, set at 37°C, with an atmosphere containing 5% CO_2_ and 95% humidity.

To assess the efficacy of both extracts in our cell culture models, cells were plated and grown to 50–70% confluence prior to treatment with STE or RE, at increasing concentrations ranging from 0.01 mg/mL to 0.25 mg/mL. After treatment, cells were analyzed for the efficacy of STE and RE, as described below. All cells were cultured for ≤4 months, before being discarded, and fresh frozen cells were used to continue studies, lasting longer than the 4 months period.

### 2.3. Assessment of Programmed Cell Death Induction

Annexin V binding assay and propidium iodide staining were performed to, respectively, monitor early apoptosis and cell permeabilization, a marker of necrotic or late apoptotic cell death. Cells were washed with phosphate buffer saline (PBS) and suspended in Annexin V binding buffer (10 mM of HEPES, 140 mM of NaCl, 2.5 mM of CaCl2, and pH of 7.4) with green fluorescent Annexin V Alexa Fluor 488 (1 : 20) (Life Technologies Inc, Cat. no. A13201, Burlington, ON, Canada) and 0.01 mg/mL of red fluorescent PI (Life Technologies Inc, Cat. no. P3566, Burlington, ON, Canada) for 15 minutes at 37°C and protected from light. The percentage of early (green) and late apoptotic cells (green and red) and necrotic cells (red) was quantified with a Tali Image-Based Cytometer (Life Technologies Inc., Cat. no. T10796, Burlington, ON, Canada). Cells from at least 13 random fields were analyzed by using both the green (ex. 458 nm; em. 525/20 nm) and red (ex. 530 nm; em. 585 nm) channels. Fluorescent micrographs were taken at 200x or 400x magnification using LAS AF6000 software with a Leica DMI6000 fluorescent microscope (Wetzlar, Germany). Cells monitored with microscopy were counterstained with Hoechst 33342 (Molecular Probes, Eugene, OR, USA) with a final concentration of 10 *μ*M following the 15-minute incubation. The protocol used is similar to that of previously published work [31].

### 2.4. Assessment of Cellular Activity and Viability

To examine the viability of breast cancer cells after treatment, cells were incubated for 48 hours following the desired treatments and then incubated once more with cell proliferation reagent WST-1 (Catalogue no. 05 015 944 001, Roche Diagnostics) for 3 hours at 37°C. Absorbance readings of the formazan product were obtained at 450 nm by using a spectrofluorometer (SpectraMax Gemini XS, Molecular Devices, Sunnyvale, CA). Viability readings were analyzed using GraphPad Prism 6.0 software and expressed as a percentage of the control untreated groups.

### 2.5. Evaluation of Mitochondrial Membrane Potential

0.1 *μ*M of tetramethylrhodamine methyl ester (TMRM; Gibco BRL; VWR; Cat. no. 89139-392) was used for detecting mitochondrial membrane potential (MMP), an indicator of healthy intact mitochondria. Following incubation with TMRM, cells were collected, washed with 1 × PBS, resuspended in PBS, and then analyzed using the Tali Image-Based Cytometer (Life Technologies Inc; Cat. no. T10796). Cells from 13 random fields were analyzed by using the red (ex = 530 nm; em = 585 nm) channel. Fluorescent micrographs were taken at 200x or 400x magnification by using LAS AF6000 software with a Leica DMI6000 fluorescent microscope (Wetzlar, Germany). Cells monitored with microscopy were stained with 0.1 *μ*M of TMRM and counterstained with 10 *μ*M of Hoechst 33342 (Molecular Probes, Eugene, OR, USA). The protocol used is similar to that of the previously published work [31].

### 2.6. Quantification and Inhibition of Reactive Oxygen Species (ROS)

Whole-cell ROS generation was monitored with the small molecule 2′, 7′-dichlorofluorescein diacetate (H2DCFDA). H2DCFDA enters the cell and is deacetylated by esterases and oxidized by ROS to the highly fluorescent 2′, 7′-dichlorofluorescein (DCF) (excitation 495 nm; emission 529 nm). Cells were pretreated with 20 *μ*M of H2DCFDA (Sigma-Aldrich Canada, Cat. no. D6883, Mississauga, ON, Canada) for 30 min at 37°C protected from light at 5% CO_2_.

Cells were treated for the indicated durations, collected, centrifuged at 3500 × *g* for 5 min, and resuspended in PBS. The percentage of DCF-positive cells was quantified by using the Tali Image-Based Cytometer (Life Technologies Inc., Burlington, ON, CA, Cat no. T10796) using 13 random fields per group with the green channel (excitation: 458 nm; emission: 525/20 nm).

N-acetylcysteine (NAC) was used to evaluate the effect of ROS inhibition on the apoptosis induction of the extracts. Cells were treated with varying concentrations of both extracts with or without NAC (5 mM per well), a known inhibitor of ROS, and then incubated for 48 hours. The cell viability was assessed by using the WST-1 viability assay as described in Section 2.4.

Fluorescent micrographs were taken at 200x or 400x magnification using LAS AF6000 software with a Leica DMI6000 fluorescent microscope (Wetzlar, Germany). Cells monitored with microscopy were stained with 20 *μ*M of H2DCFDA and counterstained with 10 *μ*M of Hoechst 33342 (Molecular Probes, Eugene, OR, USA). The protocol used is similar to that of previously published work [31].

### 2.7. Assessment of Cell Viability by Analyzing Cell Morphology

Brightfield images were taken at 200x or 400x magnification by using ToupLite software with the OMAX A35100U Microscope. The images were used to monitor treatment groups for apoptotic features, such as cell shrinkage, membrane blebbing, and nuclear condensation.

### 2.8. Wound Healing Assay

MDA-MB-231 cells were grown to 80% confluency in 6-well tissue culture plates. Cells were scratched with a sterile P200 pipette tip to create a cell-free gap. Loosely attached cells were removed by PBS washing. Cells were treated with 0.1 mg/mL of STE, 0.50 *μ*M of cisplatin, 0.01 *μ*M of Taxol, and both herb-drug combinations. Cells in the negative control received no treatment. The progression of migration was photographed at 96 hours under fluorescence and bright-field microscopy following staining with Hoechst 33342 (Molecular Probes, Eugene, OR, USA). The wound healing closure distance by the migrated cells was measured and averaged over four independent experiments for each treatment group. The protocol used is similar to that of previously published work [31].

### 2.9. Immunofluorescence Staining for Cancer Stem Cells

Cells were treated with 0.50 *μ*M of cisplatin, 0.25 mg/mL of STE combined with 0.1 mg/mL of RE, and different combinations of the extracts with the drugs cisplatin + RE, cisplatin + STE, cisplatin + RE + STE, taxol + RE, taxol + STE, and taxol + STE + RE. Cells were plated in 8-well tissue culture plates and stained with the standard immunofluorescence protocol. In short, cells were washed with 1x PBS and fixed with 3.7% formaldehyde for 15 minutes. Cells were then washed with 1x PBS and permeabilized with 0.15% Triton X-100 (Sigma-Aldrich, USA) for 2 minutes. Cells were washed with tris-buffered saline (TBS), blocked with 5% bovine serum albumin (BSA) (BioShop Canada Inc., Canada), incubated on a rocker for one hour, and again washed with TBS. A 1 : 1000 dilution of the primary antibody anti-mouse CD44 (obtained from Abcam, Canada, catalogue number ab6124) was prepared in TBS and added to each well, and cells were incubated on a rocker for another hour. Cells were then washed with TBS and incubated on the rocker for 5 minutes three times. A 1 : 1000 dilution of the secondary antibody Alexa Fluor 488 donkey anti-mouse IgG (H + L) (Life Technologies Corporation, Oregon, catalogue number A10042) was prepared in TBS and added to each well, and cells were incubated on a rocker for another hour. Cells were again washed with TBS and incubated on the rocker for 5 minutes three times. Cells were then stained with Hoechst 33342 (Molecular Probes, Eugene, OR, USA). Slides were preserved with 50% glycerol (Sigma-Aldrich, Germany), and images were visualized using LAS AF6000 software with a Leica DMI6000 fluorescent microscope (Wetzlar, Germany). The protocol used is similar to that of previously published work [23].

### 2.10. Statistical Analysis

Statistical analyses of wound healing assays and immunofluorescence staining for cancer stem cells were performed in R (R Core Team, 2022) by using a one-way analysis of variance (ANOVA) and Tukey's multiple comparison of means. All other statistical analyses were performed by using a two-way ANOVA in GraphPad Prism version 6.0 for Windows, GraphPad Software, San Diego, California, USA (https://www.graphpad.com). All trials were conducted for at least three independent times.

## 3. Results

### 3.1. Synthite Tea and Rosemary Extracts Reduce Viability of Triple-Negative Breast Cancer Cells

**Figure 1:**
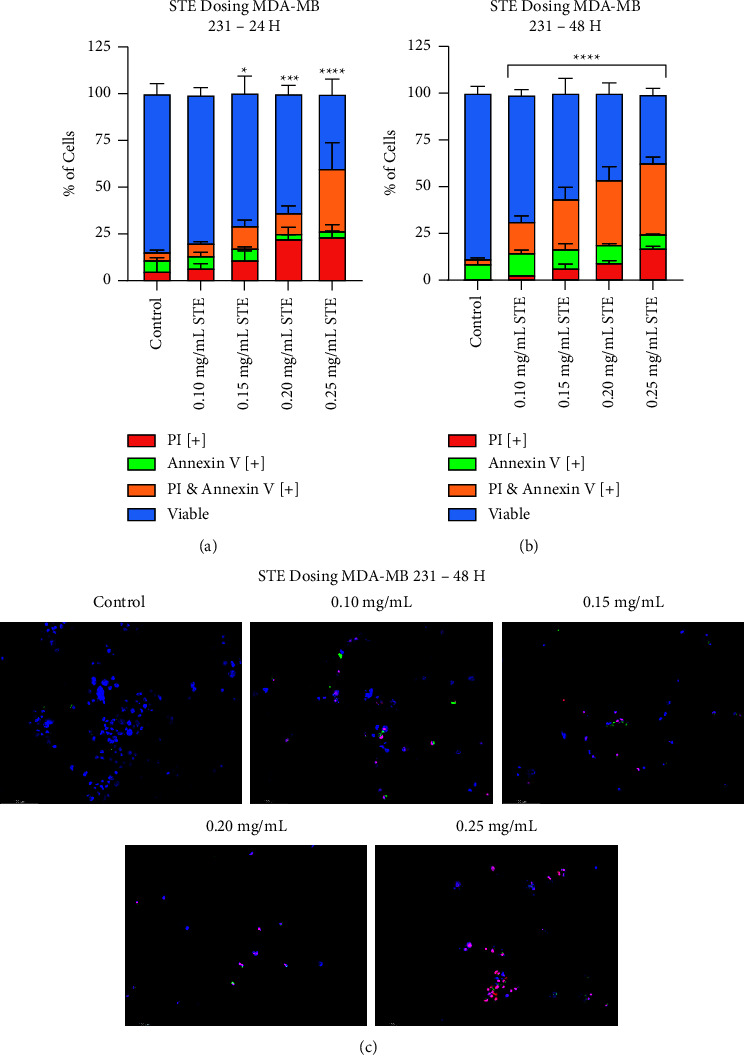
Synthite tea extract induces apoptosis in breast cancer in a dose-dependent and time-dependent manner. MDA-MB-231 cells were treated with various concentrations of STE at (a) 24 hours and (b) 48 hours. The graphed results were obtained by using image-based cytometry to assess the percentage of cells positive with fluorescence associated with Annexin V (green), PI (red), and both (yellow) or negative for both Annexin V and PI (blue). Values are expressed as a mean ± SD from three independent experiments. STE treatment groups are compared with a negative control of ddH_2_O. Brightfield images were taken at 200x or 400x magnification using ToupLite software with the OMAX A35100U Microscope. Images are representative of the three independent experiments. Statistical calculations were performed by using a two-way ANOVA multiple comparison test. ^*∗*^*p* < 0.05 vs control, ^*∗∗*^*p* < 0.01 vs control, ^∗∗∗^ *p* < 0.001 vs the control, and ^*∗∗∗∗*^*p* < 0.0001 vs control. (c) Fluorescent microscopy of STE apoptosis induction in MDA-MB-231 cells at 48 hours. Fluorescent images were stained with Annexin V (green), PI (red), and Hoechst (blue) at 200x magnification. Scale bar is 100 microns. Images are representative of three independent experiments.

**Figure 2:**
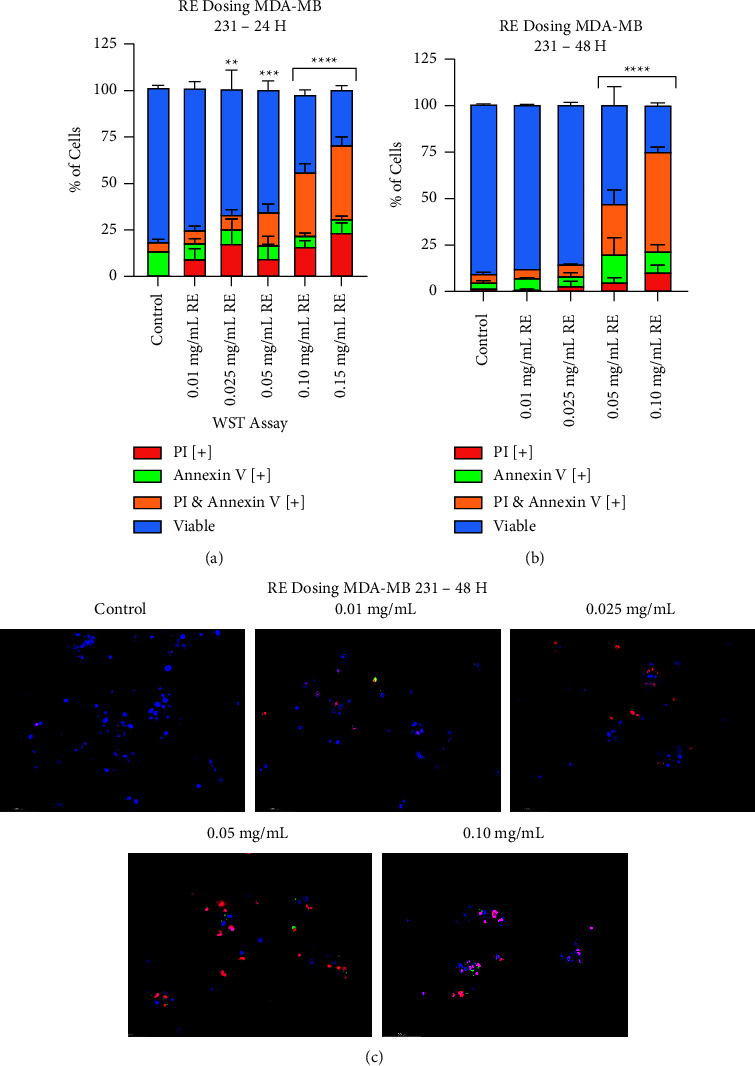
Rosemary extract induces apoptosis in breast cancer in a dose-dependent manner. MDA-MB-231 cells were treated with various concentrations of RE at (a) 24 hours and (b) 48 hours. The graphed results were obtained by using image-based cytometry to assess the percentage of cells positive with fluorescence associated with Annexin V (green), PI (red), and both (yellow) or negative for both Annexin V and PI (blue). Values are expressed as a mean ± SD from three independent experiments. RE treatment groups are compared with a negative control of DMSO. Brightfield images were taken at 200x or 400x magnification using ToupLite software with the OMAX A35100U microscope. Images are representative of three independent experiments. Statistical calculations were performed by using a two-way ANOVA multiple comparison test. ^*∗*^*p* < 0.05 vs control, ^*∗∗*^*p* < 0.01 vs control, ^∗∗∗^ *p* < 0.001 vs the control, and ^*∗∗∗∗*^*p* < 0.0001 vs control. (c) Fluorescent microscopy of RE apoptosis induction in MDA-MB-231 cells at 48 hours. Fluorescent images were stained with Annexin V (green), PI (red), and Hoechst (blue) at 200x magnification. Scale bar is 100 microns. Images are representative of three independent experiments.

This work initially assessed the anticancer activity of each extract as a standalone therapeutic against MDA-MB-231 breast cancer cells. To determine the apoptosis-inducing capabilities of both STE and RE, an Annexin V and propidium iodide (AVPI) assay was used. The cells were stained with the fluorescent apoptotic-marking dyes, Annexin V (AV) and propidium iodide (PI). AV binds to phosphatidylserine once it has externalized onto the outer membrane, a hallmark of apoptosis. Meanwhile, PI is a DNA intercalating dye that will only permeabilize with apoptotic cells. The absence of AV or PI staining represents viable cells, as shown in the graph legend. On the left of Figures 1(a), 1(b), 2(a), and 2(b), the percentage of apoptotic cells is graphed.

Concentrations ranging from 0.10 mg/mL to 0.25 mg/mL of aqueous STE were tested at 24 (Figure 1(a)) and 48 hours (Figure 1(b)). At 24 hours, concentrations of 0.15 mg/mL of STE and onward significantly induced apoptosis when compared to the negative control. At 48 hours, every tested concentration of STE showed statistically significant apoptosis induction. Therefore, the extract displays clear time and dose dependency.

An analysis of cell morphology confirms these results at 24 (Figure 1(a)) and 48 hours (Figure 1(b)). The control group is characterized by long, spindle-like shapes and the absence of apoptotic features, such as cell shrinkage, membrane blebbing, and nuclear condensation. However, as the concentration of the extract increased, the cells distinctly differed with the control group's morphology. STE treatment groups are characterized by the previously mentioned apoptotic markers, most evidently in the 0.25 mg/mL group.

Similarly, concentrations of 0.01 mg/mL–0.10 mg/mL of RE were tested at 24 (Figure 2(a)) and 48 hours (Figure 2(b)). At 24 hours, 0.01 mg/mL did not induce significant apoptosis, but 0.025 mg/mL and onwards did. A similar trend was observed at 48 hours, where all concentrations aside from 0.01 mg/mL induced apoptosis. An analysis of cell morphology (Figures 2(a) and 2(b)) may confirm these results. The RE treatment groups clearly displayed apoptotic morphology. Interestingly, the 0.025 mg/mL treatment group induced less apoptosis at 48 hours than at 24 hours, suggesting that its efficacy diminished over time.

Fluorescent microscopy (Figures 1(c) and 2(c)) served as supplementary qualitative evidence in support of the AVPI assays. Cells were once again stained with AV (green) and PI (red) prior to treatment with STE and then photographed at 48 hours. In addition, cells were counterstained with Hoechst (blue). All cells, viable or apoptotic, stained blue, while only apoptotic cells stained red and/or green. As shown by the increase in red and green fluorescence, STE and RE significantly induced apoptosis in triple-negative breast cancer with dose dependency (Figures 1(c) and 2(c), respectively).

### 3.2. Investigating STE and RE's Interactions with Common Chemotherapeutics: Herb-Herb and Herb-Drug Interactions

Once again, the apoptosis-inducing effects of STE and RE were determined by using the AVPI assay along with brightfield and fluorescent microscopy. MDA-MB-231 cells were treated with varying combinations of STE (0.10 mg/mL), RE (0.025 mg/mL), cisplatin (0.50 *µ*M), and Taxol (0.01 *µ*M).

**Figure 3:**
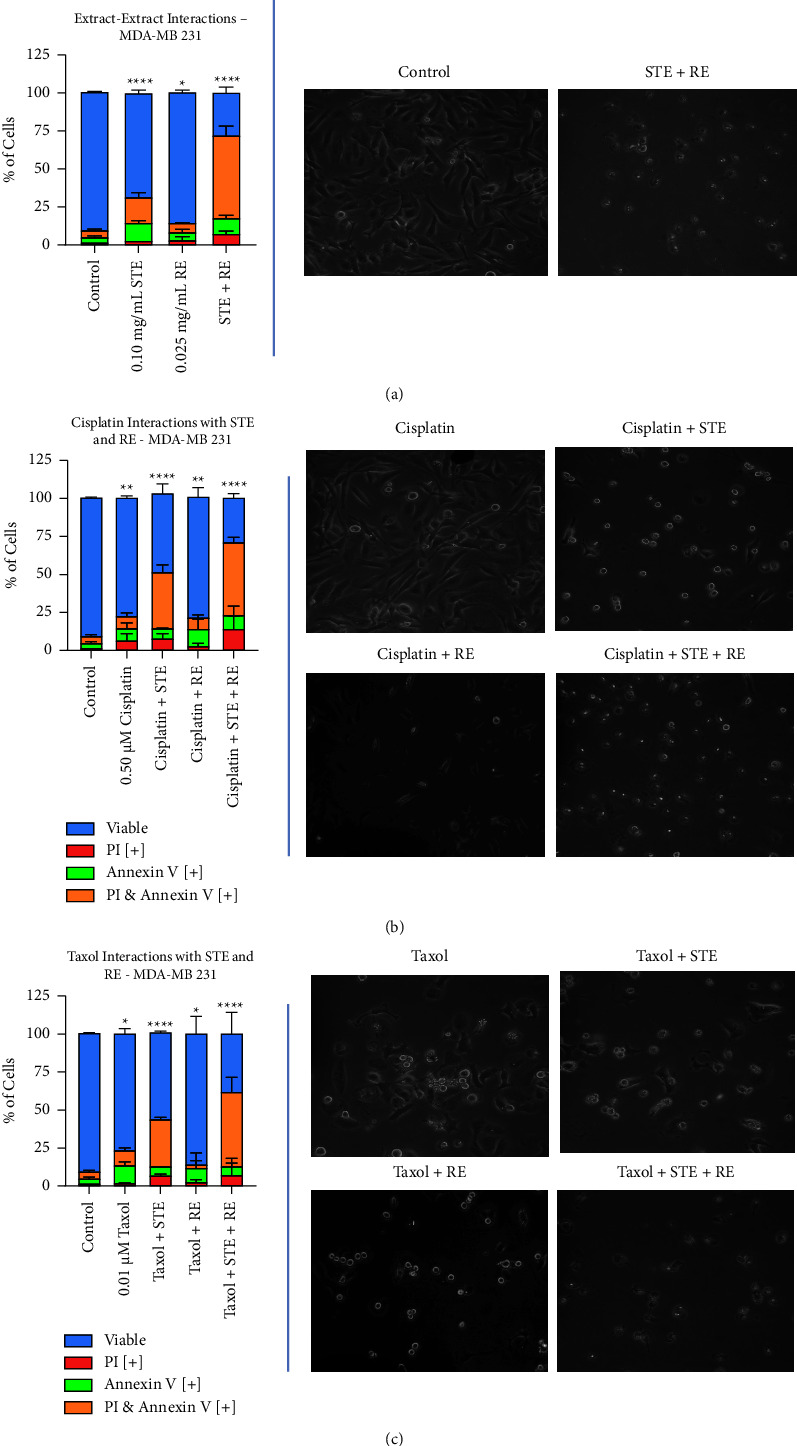
Synthite tea and rosemary extracts positively interacted with each other and with chemotherapeutics in triple-negative breast cancer. MDA-MB-231 cells were treated with varying combination therapies: (a) 0.10 mg/mL of Synthite tea and 0.025 mg/mL of rosemary, (b) 0.50 *µ*M of cisplatin on its own and in combination with both extracts, and (c) 0.01 *µ*M of Taxol on its own and in combination with both extracts. The graphed results were obtained by using image-based cytometry to assess the percentage of cells positive with fluorescence associated with Annexin V (green), PI (red), and both (yellow) or negative for both Annexin V and PI (blue). Values are expressed as a mean ± SD from three independent experiments. All treatment groups are compared with a negative control of DMSO. ^*∗*^*p* < 0.05 vs control, ^*∗∗*^*p* < 0.01 vs control, and ^*∗∗∗∗*^*p* < 0.0001 vs control.

**Figure 4:**
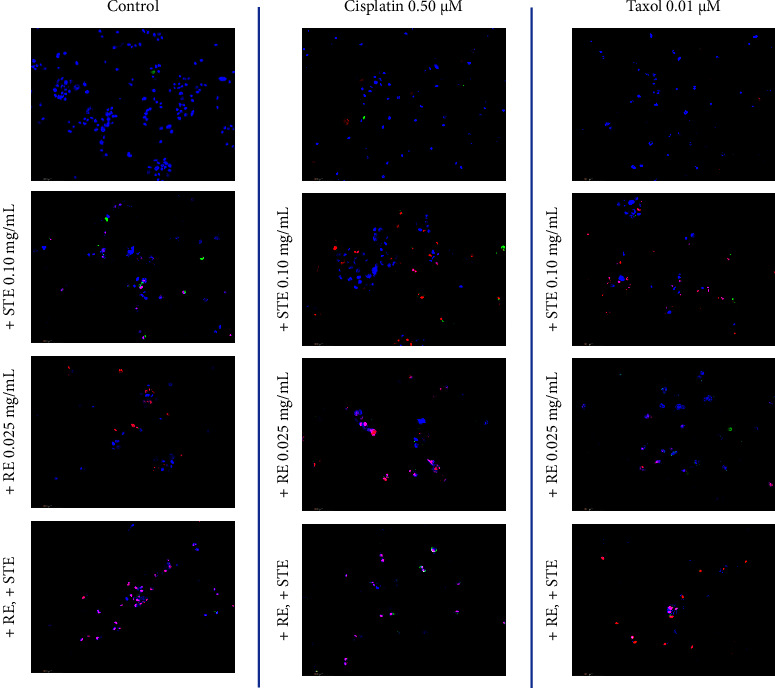
Fluorescent micrographs of varying combination therapies in triple-negative breast cancer at 48 hours. MDA-MB-231 micrographs of various herb-herb and herb-drug interactions at 48 hours. Fluorescent images were stained with Annexin V (green), PI (red), and Hoechst (blue) at 200x magnification. Scale bar is 100 microns. Images are representative of three independent experiments.

According to Figures 3(a) and 3(d), STE and RE are significantly much more effective when administered in combination. Results from the AVPI assay (Figure 4(a)) suggest a synergistic effect, as the combination treatment reduced cell viability more than either extract did on its own. The brightfield images confirm these findings, as the cells have significantly shrunk and condensed.

As shown in Figures 3(b) and 3(c), Synthite tea positively interacted with cisplatin and Taxol. Combining relatively low doses of both STE and cisplatin resulted in a great reduction in cell viability when compared to either component on its own. As well, STE enhanced Taxol's anticancer activity. The STE-Taxol combination group induced more apoptosis than Taxol did on its own but was not found to be statistically different than STE on its own. Provided on the right in figures Figures 4(b) and 4(c), there is a distinct loss in healthy cellular morphology in the STE-drug combination groups.

On the other hand, rosemary did not interact in any manner with either chemotherapy. The RE-cisplatin group did not result in more cell death than RE or cisplatin did on their own. In the same vein, the RE-Taxol group was also not found to have better anticancer activity than either RE or Taxol did on their own. The brightfield images (Figures 3(b) and 3(c)) only partially agree with AVPI results. Contrary to the AVPI graph, there is a significant increase in apoptotic features in the cisplatin-RE group when compared to cisplatin on its own. Moving on, the Taxol-RE image shows no discernible difference between Taxol and Taxol-RE, as expected.

Finally, the herb-herb-drug groups induced significant cell death in breast cancer. In Figures B and D, we see that cisplatin-STE-RE was much more effective than the cisplatin-STE and the cisplatin-RE groups were. As well, the Taxol-STE-RE group induced more cell death than the STE-Taxol and RE-Taxol groups. The micrographs on the right show that in both herb-herb-drug groups, the cells have lost all resemblance to the control group. Most importantly, as shown in Figure 4(d), neither of the two STE-RE-chemotherapy groups was more effective than the STE-RE group.

**Figure 5:**
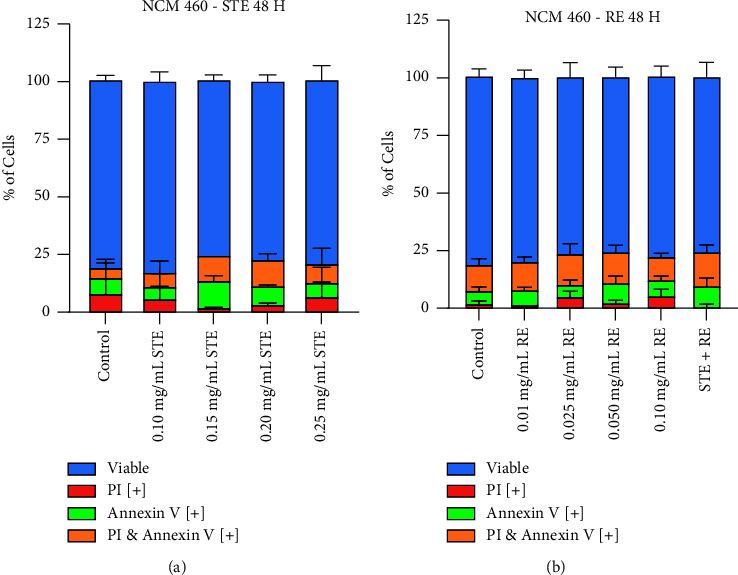
Synthite tea and rosemary extracts are both selective for breast cancer cells. NCM-460 cells were treated with varying concentrations of (a) STE and (b) RE (0.01 mg/mL–0.25 mg/mL) for 48 hours. Results were obtained by using image-based cytometry to assess the percentage of cells positive with fluorescence associated with Annexin V (green), PI (red), and both (yellow) or negative for both Annexin V and Pi (blue). Values are expressed as a mean ± SD from three independent experiments. ^*∗*^*p* < 0.05 vs control, ^*∗∗*^*p* < 0.01 vs control, and ^*∗∗∗∗*^*p* < 0.0001 vs control.

Fluorescent images of STE, RE, cisplatin, Taxol, and all their combinations are provided in Figure 5. As the chemotherapeutics were administered at low doses, they did not induce much apoptosis, hence the low amount of red and green fluorescence. However, as the chemotherapies were supplemented with STE or RE, there appears to be more cell death. Finally, when the chemotherapies were combined with both the extracts, almost every single cell underwent PCD as they were stained in either red or green. The fluorescent micrographs are in accordance with the results in Figure 4. The extracts positively interacted with each other and with conventional chemotherapeutics.

The statistical significance of cell viability was analyzed for every combination therapy and its individual components. Compared to the control, cell viability was significantly reduced in cells treated with 0.10 mg/mL of STE (*p* < 0.0001), 0.025 mg/mL of RE (*p* < 0.05), and a combination of STE + RE (*p* < 0.0001). Treatment with 0.10 mg/mL of STE + 0.025 mg/mL of RE significantly reduced cell viability compared to treatment with either extract alone (*p* < 0.00001 for both comparisons).

Cell viability was significantly reduced compared to the control in cells treated with 0.50 *µ*M of cisplatin (*p* < 0.01), cisplatin combined with 0.10 mg/mL of STE (*p* < 0.0001), cisplatin combined with 0.025 mg/mL of RE (*p* < 0.01), and cisplatin combined with both extracts (*p* < 0.0001). Compared to cells treated with cisplatin alone, cell viability was significantly reduced in cells treated with cisplatin + STE (*p* < 0.00001) and cisplatin + STE + RE (*p* < 0.00001). There was no significant difference in cell viability in cells treated with cisplatin + RE compared to cells treated with cisplatin alone and cells treated with RE alone. Cell viability was significantly reduced in cells treated with cisplatin + STE + RE compared to cells treated with cisplatin alone (*p* < 0.00001), STE alone (*p* < 0.00001), and RE alone (*p* < 0.00001), STE + cisplatin (*p* < 0.00001), and RE + cisplatin (*p* < 0.00001). There was no significant difference in cell viability between cells treated with cisplatin + STE + RE and cells treated with STE + RE.

Cell viability was significantly reduced compared to the control in cells treated with 0.01 *µ*M of Taxol (*p* < 0.05), Taxol combined with 0.10 mg/mL of STE (*p* < 0.0001), Taxol combined with 0.025 mg/mL of RE (*p* < 0.05), and Taxol combined with both extracts (*p* < 0.0001). Compared to cells treated with Taxol alone, cell viability was significantly reduced in cells treated with Taxol + STE (*p* < 0.00001). There was no significant difference in cell viability in cells treated with STE + Taxol compared to cells treated with STE alone. There was no significant difference in cell viability in cells treated with Taxol + RE compared to cells treated with Taxol alone and cells treated with RE alone. Cell viability was significantly reduced in cells treated with Taxol + STE + RE compared to cells treated with Taxol alone (*p* < 0.00001), STE alone (*p* < 0.00001), and RE alone (*p* < 0.00001), STE + Taxol (*p* < 0.01), and RE + Taxol (*p* < 0.00001). There was no significant difference in cell viability between cells treated with Taxol + STE + RE and cells treated with STE + RE.

### 3.3. Synthite Tea and Rosemary Extracts Do Not Induce Apoptosis in Noncancerous Cells

The selectivity of both Synthite tea (Figure 5(a)) and rosemary (Figure 5(b)) extracts was assessed by using the noncancerous cell line, NCM-460. Varying doses of both extracts were individually administered and assessed at 48 hours. The Annexin V and propidium iodide (AVPI) assay was used to determine whether these NHPs had adverse effects on noncancerous cells. The cells were stained with the fluorescent apoptotic-marking dyes, Annexin V (AV) and propidium iodide (PI).

None of the tested concentrations were significantly different compared to the control, and so none of the administered concentrations induced apoptosis in noncancerous cells. Therefore, we have demonstrated that both NHPs induce cancer-specific apoptosis, leaving normal cells unaffected.

### 3.4. Assessment of Anticancer Activity of STE, RE, Chemotherapeutics, and Combinations with Wound Healing Assay

**Figure 6:**
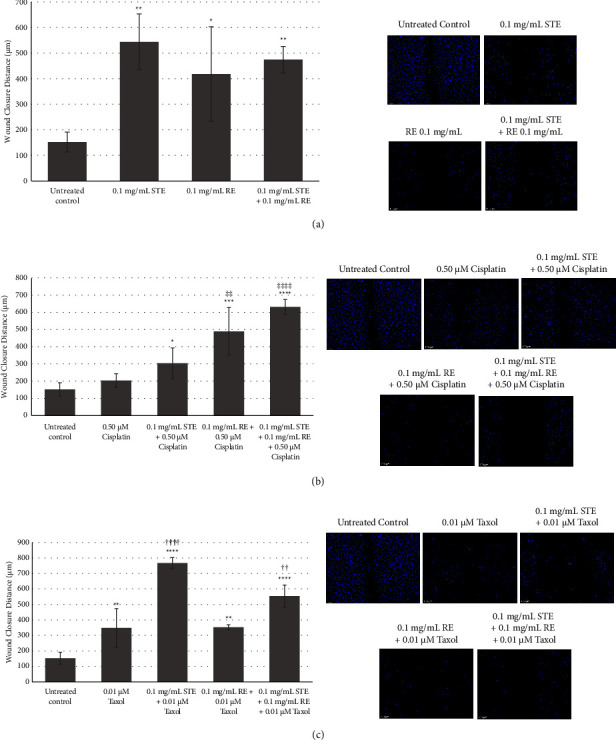
Wound healing assay for anticancer activity of Taxol, cisplatin, STE, RE, and combinations at 96 hours. Wound healing assay at 96 hours following treatment with (a) STE, RE, and combination; with (b) cisplatin and cisplatin-extract combinations; with (c) Taxol and Taxol-extract combinations. Bars represent the average wound closure distance for each group (*n* = 4) in MDA-MB-231 cells. Error bars represent the positive and negative standard deviation values of each group. ^*∗*^*p* < 0.05, ^*∗∗*^*p* < 0.01, ^*∗∗∗*^*p* < 0.001, and ^*∗∗∗∗*^*p* < 0.0001 compared to the control. ^‡^*p* < 0.05, ^‡‡^*p* < 0.01, ^‡‡‡^*p* < 0.001, and ^‡‡‡‡^*p* < 0.0001 compared to cisplatin alone. ^†^*p* < 0.05, ^††^*p* < 0.01, ^†††^*p* < 0.001, and ^††††^*p* < 0.0001 compared to Taxol alone. Micrographs represent the overlay of fluorescence and brightfield images at 100x magnification. Scale bar is 100 *μ*m. Each micrograph is representative of four independent experiments.

Compared to the untreated control, wound closure distance was significantly greater in cells treated with 0.1 mg/mL of STE alone (*p* < 0.01), 0.1 mg/mL of RE alone (*p* < 0.05), and 0.1 mg/mL of STE and 0.1 mg/mL of RE combined (*p* < 0.01) (Figure 6(a)).

The average wound closure distance following treatment with cisplatin and cisplatin-extract combinations is shown in Figure 6(b). Wound closure distance in cells treated with cisplatin alone was not significantly different than that of the negative control. Compared to the untreated control, wound closure distance was significantly greater in cells treated with 0.1 mg/mL of STE and 0.50 *μ*M of cisplatin combined (*p* < 0.05) and 0.1 mg/mL of RE and 0.50 *μ*M of cisplatin combined (*p* < 0.001), as well as 0.1 mg/mL STE, 0.1 mg/mL RE, and 0.50 *μ*M cisplatin combined (*p* < 0.0001). Wound closure distance was significantly greater in cells treated with the RE-cisplatin combination (*p* < 0.01) and the STE-RE-Taxol combination (*p* < 0.0001) compared to cells treated with cisplatin alone.

The average wound closure distance following treatment with Taxol and Taxol-extract combinations is shown in Figure 6(c). Treatment with 0.01 *μ*M of Taxol alone (*p* < 0.01), 0.1 mg/mL of STE and 0.01 *μ*M of Taxol combined (*p* < 0.0001), and 0.1 mg/mL of RE and 0.01 *μ*M of Taxol combined (*p* < 0.01), as well as 0.1 mg/mL of STE, 0.1 mg/mL of RE, and 0.01 *μ*M of Taxol combined (*p* < 0.0001) significantly increased the wound closure distance compared to the untreated control. Wound closure distance was significantly greater in cells treated with the STE-Taxol combination (*p* < 0.0001) and the STE-RE-Taxol combination (*p* < 0.01) compared to cells treated with Taxol alone.

Average wound closure distance was significantly greater in cells treated with STE alone compared to cells treated with Taxol alone (*p* < 0.05) and compared to cells treated with cisplatin alone (*p* < 0.01). There was no significant difference in wound closure distance in cells treated with RE and cells treated with either chemotherapeutic alone. Wound closure distance in cells treated with the STE-RE combination was significantly greater than that of the cells treated with cisplatin alone (*p* < 0.01) but did not differ from Taxol alone.

### 3.5. The Induction of ROS Production in Breast Cancer

**Figure 7:**
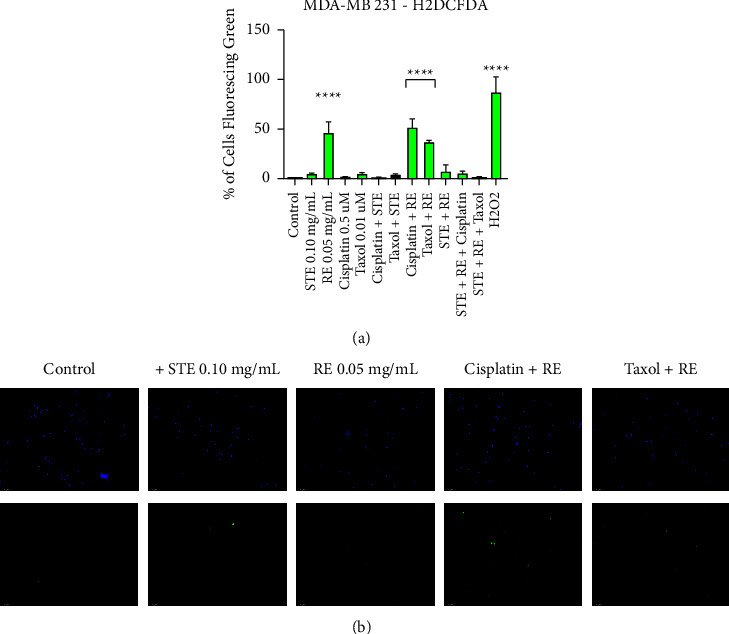
RE induces ROS production in triple-negative breast cancer. (a) MDA-MB-231 breast cancer cells were treated with H2DCFDA following treatments with Taxol and cisplatin individually and in combination with STE and RE. Results were obtained by using image-based cytometry to assess the percentage of cells positive with DCF, which indicates the generation of ROS. Hydrogen peroxide served as a positive control. Treatment groups were compared with the negative control DMSO. Values are expressed as a mean ± SD from three independent experiments. Statistical calculations were performed by using a two-way ANOVA multiple comparison test. ^*∗*^*p* < 0.05 vs control, ^*∗∗*^*p* < 0.01 vs control, and ^*∗∗∗∗*^*p* < 0.0001 vs control. (b) Fluorescent microscopy of triple-negative breast cancer cells stained with H2DCFDA (green) and counterstained with Hoechst (blue) at 200x magnification. Cells fluorescing green indicate the generation of ROS. Images are representative of three independent experiments.

The introduction outlines the importance of ROS as cancer-specific targets for NHPs. The H2DCFDA assay (Figure 7(a)) was used to further characterize the effects that RE and STE have on oxidative stress in breast cancer. We observed that 0.05 mg/mL of rosemary extract induced the production of ROS in MDA-MB-231 cells. In contrast, Synthite tea extract displayed minimal ROS production and was not observed to be statistically different than the negative control group. The RE-chemo groups induced the same levels of ROS as the RE standalone treatment. Therefore, there are neither enhancing nor inhibitory effects on the extract's ROS production when combined with standard chemotherapeutics. However, when RE was combined with STE, its ROS-inducing activity was no longer observed.

Fluorescent microscopy (Figure 7(b)) confirms the results found in Figure 7(a). Green fluorescence indicates the production of ROS, while all cells will stain blue. On the top is the blue channel on its own, while the bottom is the green channel on its own. When compared with the control group, the rosemary treatment groups have much more green fluorescence. Meanwhile, the STE treatment showed minimal to no green fluorescence. Therefore, RE induces ROS in breast cancer while STE does not.

### 3.6. The Dependence on Oxidative Stress to Induce Apoptosis

**Figure 8:**
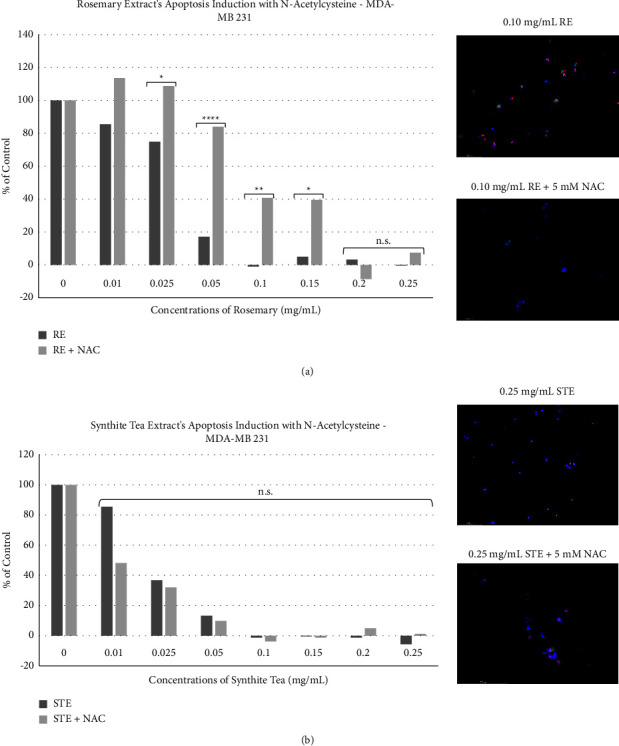
RE is dependent on the production of oxidative stress to induce apoptosis. The addition of N-acetylcysteine (NAC), a known ROS inhibitor, reduced rosemary-induced apoptosis in breast cancer. MDA-MB-231 was treated with concentrations ranging from 0.01 mg/mL to 0.25 mg/mL of both (a) RE with or without NAC and (b) STE with or without NAC. Treatment groups were compared with the negative control DMSO. Graphed results (left) concerning cell viability were obtained using the WST-1 assay. MDA-MB-231 micrographs of STE and RE with or without NAC at 48 hours (right). Absorbance readings of the formazan product were obtained at 450 nm by using a spectrofluorometer. Values are expressed as a mean ± SD from three independent experiments. Statistical calculations were performed by using a two-way ANOVA multiple comparison test. ^*∗*^*p* < 0.05 vs control, ^*∗∗*^*p* < 0.01 vs control, and ^*∗∗∗∗*^*p* < 0.0001 vs control. Fluorescent images were stained with Annexin V (green), PI (red), and Hoechst (blue) at 200x magnification. Scale bar is 100 microns. Images are representative of three independent experiments.

**Figure 9:**
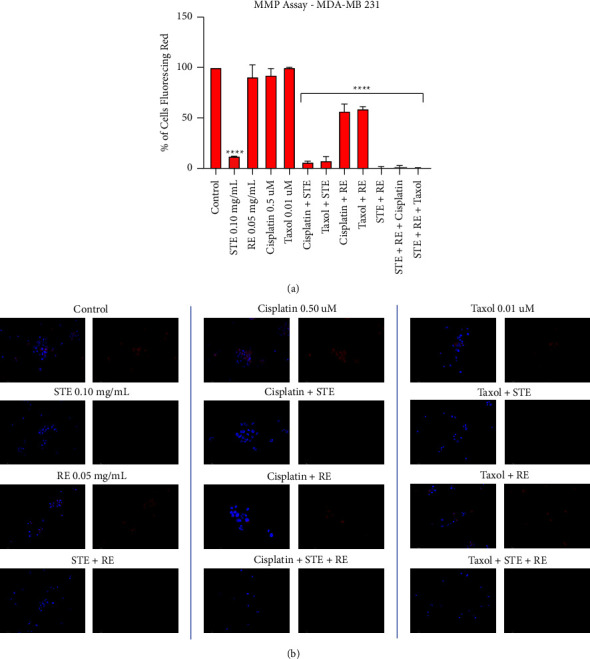
STE is dependent on the depolarization of cancerous mitochondrial membrane potential to induce apoptosis. (a) MDA-MB-231 cells were treated with chemotherapeutics Taxol and cisplatin individually and in combination with STE and RE and assessed at 48 hours. Results were obtained by using image-based cytometry to assess the percentage of cells positive with fluorescence associated with mitochondrial membrane potential (TMRM, fluoresces red). Treatment groups were compared with the negative control DMSO. Values are expressed as a mean ± SD from three independent experiments. ^*∗*^*p* < 0.05 vs control, ^*∗∗*^*p* < 0.01 vs control, and ^*∗∗∗∗*^*p* < 0.0001 vs control. (b) Fluorescent microscopy of triple-negative breast cancer cells stained with TMRM (red) and counterstained with Hoechst (blue) at 200x magnification. Overlays of both fluorescent channels are on the left, and the red channel alone is on the right. Images are representative of three independent experiments.

As outlined in Section 3.3, only one of the extracts, rosemary, was found to induce ROS in breast cancer. In this section, we investigate the effect that ROS has on rosemary's ability to induce apoptosis. Cells were pretreated with N-acetylcysteine (NAC), a known inhibitor of oxidative stress. Then, cells were treated with either STE or RE at concentrations ranging from 0.01 mg/mL to 0.25 mg/mL. As shown in Figure 8(a), the addition of NAC significantly inhibited RE's apoptosis induction in breast cancer. The majority of RE concentrations (0.025 mg/mL–0.15 mg/mL) were affected by the addition of NAC, while the highest doses were not. In contrast, STE's anticancer activity was completely unaffected by NAC (Figure 8(b)). These findings were confirmed with fluorescent microscopy (Figures 9(a) and 9(b)). In the micrographs of 0.10 mg/mL of RE, there is significant red and green fluorescence, indicating interactions with Annexin V and PI apoptotic markers. Yet, when NAC is added, there is a minimal green or red fluorescence. Furthermore, the micrographs in Figure 8(b) show that the red and green fluorescence displayed by the STE treatment group is similar to that of the STE supplemented with NAC. As is consistent with the results in Section 3.3, RE is dependent on the induction of ROS to induce apoptosis in breast cancer, while STE is not.

### 3.7. Mitochondrial Depolarization

Cancerous mitochondria serve as attractive targets for therapies that aim to specifically target cancer. Mitochondrial dysfunction may ultimately lead to the initiation of programmed cell death. The fluorescent TMRM assay was used to monitor mitochondrial stability and depolarization. Tetramethylrhodamine methyl ester (TMRM) served to quantify mitochondrial stability. TMRM will bind with healthy mitochondria; therefore, high levels of red fluorescence indicate intact and stable mitochondria. Cells were pretreated with TMRM and then treated with STE, RE, Taxol, cisplatin, and their combinations and were assessed at 48 hours. Rosemary, cisplatin, and Taxol were unable to affect MMP. In contrast, every treatment group involving STE completely collapsed the MMP to almost 0. Synthite tea showed significant destabilizing effects on cancerous mitochondria and was not inhibited by RE or either chemotherapy. Furthermore, while RE did not affect the MMP on its own, both the RE-cisplatin and RE-Taxol groups depolarized the mitochondria (Figure 9(a)).

To provide supplementary evidence, this experiment was not repeated but instead analyzed by using fluorescent microscopy (Figure 9(b)). Red fluorescence is indicative of healthy mitochondria, while its absence indicates mitochondrial depolarization. Hoechst will bind with every cell and is shown by the blue fluorescence. Each treatment group is accompanied by two side-by-side images. On the left is a merged image of both the red and blue fluorescent channels, while on the right is the red channel on its own. The control is characterized by significant red fluorescence, while every STE group had minimal red fluorescence if any at all. The RE group is comparable with the control. Therefore, the microscopy is in accordance with quantitative data, where STE acts by targeting the MMP, while RE does not.

### 3.8. Sensitivity of Cancer Stem Cells to STE, RE, Chemotherapeutics, and Combinations

**Figure 10:**
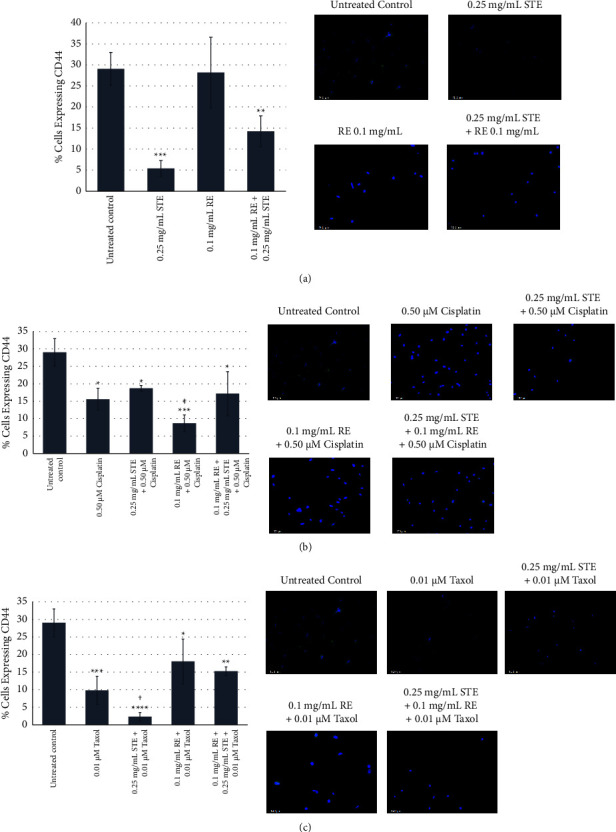
Immunofluorescence staining of stem cells with CD44 stem cell surface markers in cells treated with STE, RE, cisplatin, Taxol, and herb-drug combinations at 48 hours. Bars represent the average percentage of cells expressing CD44 for each group (*n* = 3) in MDA-MB-231 at 48 hours following treatment. Error bars represent the positive and negative SD of each group. ^*∗*^*p* < 0.05, ^*∗∗*^*p* < 0.01, ^*∗∗∗*^*p* < 0.001, and ^*∗∗∗∗*^*p* < 0.0001 compared to the control. ^‡^*p* < 0.05 compared to cisplatin alone. ^†^*p* < 0.05 compared to Taxol alone. Micrographs are overlays of fluorescence and brightfield images at 200x magnification. Scale bar is 100 *μ*m. Each micrograph is representative of the three independent experiments. Cells were stained with Hoechst 33342 (blue) and CD44 (green). (a) Immunofluorescence staining of CSCs with CD44 in cells treated with STE, RE, and a combination of STE and RE. (b) Immunofluorescence staining of stem cells with CD44 stem cell surface markers in cells treated with cisplatin and herb-cisplatin combinations. (c) Immunofluorescence staining of stem cells with CD44 stem cell surface markers in cells treated with Taxol and herb-Taxol combinations.

Compared to untreated cells, the average percentage of cells expressing CD44 was significantly less in cells treated with 0.25 mg/mL of STE (*p* < 0.001) and in cells treated with a combination of 0.1 mg/mL of RE and 0.25 mg/mL of STE (*p* < 0.01) (Figure 10(a)). The percentage of cells expressing CD44 in cells treated with 0.1 mg/mL of RE was not significantly different than the percentage in the negative control. There were significantly less cells expressing CD44 in cells treated with STE and RE compared to cells treated with RE alone (*p* < 0.05). The percentage of cells expressing CD44 was significantly less in cells treated with STE compared to cells treated with a combination of RE and STE (*p* < 0.05).

Figure 10(b) illustrates the average percentage of cells expressing CD44 following treatment with cisplatin and cisplatin-extract combinations. Compared to untreated cells, the percentage of cells expressing CD44 was significantly less in cells treated with 0.50 *μ*M of cisplatin (*p* < 0.05), 0.25 mg/mL of STE, and 0.50 *μ*M of cisplatin combined (*p* < 0.05) and 0.1 mg/mL of RE and 0.50 *μ*M of cisplatin combined (*p* < 0.001), as well as 0.25 mg/mL of STE, 0.1 mg/mL of RE, and 0.50 *μ*M of cisplatin combined (*p* < 0.05). There were significantly fewer cells expressing CD44 in cells treated with 0.1 mg/mL of RE and 0.50 *μ*M of cisplatin combined compared to cells treated with cisplatin alone (*p* < 0.05).

The average percentage of cells expressing CD44 following treatment with Taxol and Taxol-extract combinations is shown in Figure 10(c). Compared to untreated cells, there were significantly fewer cells expressing CD44 in cells treated with 0.01 *μ*M of Taxol (*p* < 0.001), 0.25 mg/mL of STE, and 0.01 *μ*M of Taxol combined (*p* < 0.0001) and 0.1 mg/mL of RE and 0.01 *μ*M of Taxol combined (*p* < 0.05), as well as 0.25 mg/mL of STE, 0.1 mg/mL of RE, and 0.01 *μ*M of Taxol combined (*p* < 0.01). Significantly fewer cells expressed CD44 when treated with 0.25 mg/mL of STE and 0.01 *μ*M of Taxol combined compared to cells treated with Taxol alone (*p* < 0.05).

## 4. Discussion

In this research paper, we present the results of the anticancer properties of STE and RE, two well-tolerated NHPs, as well as their interaction with standard chemotherapeutics. We have shown that both STE and RE induce apoptosis in triple-negative breast cancer at low doses; they enhance the anticancer activity of cisplatin and Taxol indicating positive interaction. Furthermore, these extracts with a complex mixture of bioactive compounds target the mitochondrial (STE) and oxidative vulnerability (RE) of cancer cells. Importantly, these extracts are effective in killing cancer stem cells and sensitize cancer stem cells to Taxol or cisplatin. These results demonstrate that STE and RE have the potential to be a great nontoxic supplemental therapy along with chemotherapy for triple-negative breast cancer patients and could also have prevention of relapse as they target cancer stem cells.

Induction of apoptosis by STE and RE selectively in cancer cells was clearly demonstrated by Annexin V binding assay and propidium staining in a dose-dependent manner. Purified compounds from these plant materials e.g., epigallocatechin-3-gallate in tea extract and carnosic acid, and rosmarinic acid in rosemary extract, have been shown to induce apoptosis in different cancers [40, 42]. Our hypothesis is that the total extract may contain multiple compounds that may target multiple pathways to selectively induce apoptosis in cancer cells. Additionally, the total extracts like STE and RE are characterized as natural health products that are already safe for human consumption. Most importantly, we explored the possibility of these extracts to be used in combination with chemotherapeutic drugs. Both STE and RE induced a significant percentage of apoptosis at very low doses (0.05–0.1 mg/mL). Other than the AVPI staining, the morphology of treated cells clearly indicated apoptotic features, such as nuclear condensation and blebbing. Interestingly, at similar doses, noncancerous healthy cells, normal colon mucosal cells (NCM-460), did not show any increase in apoptosis following treatment with these extracts, indicating the selective nature of these extracts for cancer cells (Figure 5).

Recently, several studies have found a variety of NHPs to have anticancer activity. However, medical professionals remain hesitant as the interactions between chemotherapeutics and NHPs are mostly uncharacterized. If NHPs should eventually be administered in clinical settings, the extracts must, at the very least, have no inhibitory effects on chemotherapeutics.

Importantly, our findings show that STE positively interacted with both cisplatin and Taxol against triple-negative breast cancer as when combined with cisplatin or Taxol, the STE-chemo group induced more apoptosis than the chemotherapeutics did on their own. Combining the chemotherapeutics with STE allows for the administration of much lower doses of the drugs while achieving even greater anticancer activity. Therefore, the herb-drug combination therapy mitigates the toxicity associated with high doses of chemotherapeutics. These findings are very important for developing STE as an adjuvant to standard chemotherapy.

The most exciting results are found in the herb-herb interaction groups. Combining 0.10 mg/mL of STE with 0.025 mg/mL of RE was, by far, the most effective combination therapy. On its own, each extract induced apoptosis in around 30% of breast cancer cells, but the combination resulted killed around 75%. Their positive interaction goes beyond an additive effect. These results indicate that a combination of these two herbal extracts may provide an alternative cancer therapy similar to or better than chemotherapeutics. Overall, we demonstrated that these nontoxic herbal extracts can be combined as a supplement to the chemo regimen where they can enhance the anticancer activity of chemotherapy. In addition, combined treatment with just the two herbal extracts has also the potential of being a very effective cancer therapy for TNBC.

The positive interaction of STE and RE with chemo was also demonstrated by wound healing assays that assesses the inhibition of proliferation and migration of cancer cells by anticancer agents [31]. While these extracts on their own were able to inhibit proliferation and migration (indicated by an increased gap in the wound compared to the control), cisplatin and Taxol on their own had very little effect. Most interestingly, a combination of the extracts with chemotherapeutics was more effective in increasing the gap width compared to either chemotherapeutic alone. These results further confirm the efficacy of the herbal extracts on their own as well as their positive interactions with chemotherapies.

Mechanistically, NHPs may achieve their selective anticancer activity by exploiting the vulnerabilities that are unique to cancerous cells. Notably, the hyperactive metabolic activity present in cancerous cells renders them susceptible to oxidative stress and mitochondrial depolarization. Indeed, some of recent research has shown these vulnerabilities in cancer cells [45]. We have demonstrated that RE's anticancer activity is reliant on the induction of ROS in breast cancer cells. We found that 0.05 mg/mL of RE induced significant production of ROS in triple-negative breast cancer. Cisplatin, Taxol, and STE did not induce any ROS on their own. However, when combined with RE, the RE-chemo groups induced ROS when compared to the control.

The important question is whether the induction of oxidative stress is critical for the induction of apoptosis by RE? We found that RE-induced apoptosis is dependent on the production of ROS as shown by the inhibition of RE-induced apoptosis by N-acetylcysteine (NAC), an antioxidant. Therefore, rosemary is dependent on the production of ROS to induce apoptosis in triple-negative breast cancer. STE treatment did not induce ROS production, and STE-induced apoptosis was not affected by NAC. Interestingly, the STE-RE group did not result in any ROS production. The lack of ROS production in the herb-herb group might be indicative of some sort of inhibitory effect between the extracts. However, the analysis of cell viability showed that STE and RE were at the very least enhancing each other, if not synergizing. One possible explanation may be that STE may be displaying protective effects related to ROS. As mentioned in the introduction, the catechins in STE are known to interact with and quench ROS *in vitro*.

Mitochondrial vulnerability of cancer cells could be also targeted by these extracts. When we assessed the effect of STE and RE on mitochondrial potential in cancer cells, we observed that STE causes mitochondrial dysfunction to induce apoptosis as indicated by the TMRM assay. We found that 0.10 mg/mL of STE completely collapsed the MMP, as there was almost 0% red fluorescence. Every combination group involving STE displayed similar results. Therefore, neither RE nor the chemotherapeutics inhibited or enhanced STE's ability to target the mitochondria.

Rosemary, cisplatin, and Taxol did not affect the mitochondria on their own. However, the RE-cisplatin and RE-Taxol groups depolarized the MMP when compared to the control. Interestingly, none of the individual components of this combination group could target the MMP, but when combined, there were depolarizing effects. This suggests that while RE cannot target the mitochondria on its own, it may potentially be sensitizing cells to mitochondrial depolarization.

As usual, fluorescent microscopy was conducted in parallel with quantitative data analysis to serve as additional evidence. In Figure 9(b), each treatment group is accompanied by two side-by-side images. On the left, both the blue and red fluorescent channels are merged together. Meanwhile, the right shows the red fluorescent channel on its own. Again, red fluorescence indicates healthy, mitochondria. The control group is characterized by significant red fluorescence, while the STE groups display minimal to no red fluorescence. In contrast, RE's red fluorescence is comparable with the control. Therefore, STE dissipated the MMP, while RE did not.

The extracts targeting different pathways is a scientifically interesting finding as it speaks on their level of selectivity. Even more, being able to target specific pathways may be useful if a cell line is found to be more resilient against certain therapies. Perhaps the synergistic effects of the STE-RE group arise from two pathways being simultaneously targeted. Further characterization of their anticancer activity will help with the scientific validation of these extracts. It is therefore important to continue characterizing the mechanisms of action of both STE and RE.

Thus, STE targets the MMP while RE targets the ROS pathway. As hypothesized, the extracts achieve selective anticancer activity by exploiting the vulnerabilities unique to cancer. Inducing ROS levels beyond the baseline that cellular protection mechanisms can cope with may initiate PCD. As explained by the Warburg effect in the introduction, the overreliance of cancerous cells on glycolysis makes their mitochondria susceptible to depolarization. STE and RE may provide a promising way to selectively target triple-negative breast cancer.

### 4.1. Sensitivity of MDA-MB-231 Stem Cells to STE and Extract-Chemotherapeutic Combinations

Cancer stem cells have been the focus of research as these cells would be responsible for cancer relapse. CD44, a stem cell surface marker, has been shown to be highly expressed in the MDA-MB-231 cell line [23, 24, 26]. We have evaluated the sensitivity of the cancer stem cells in TNBC to STE and RE. The average percentage of CSCs in control untreated MDA-MB-231 cells reported in our study (29.05% ± 3.92%) is in agreement with values previously reported in the literature [46]. Compared to untreated cells, cells treated with STE had a significantly lower percentage of CSCs after 48 h. RE on its own did not have a significant effect on CSCs. The percentage of CD44-positive cells compared to the control was significantly reduced in cells treated with cisplatin alone, cisplatin combined with STE, cisplatin combined with RE, and cisplatin combined with both extracts. The percentage of CSCs was significantly less in cells treated with RE combined with cisplatin compared to cells treated with cisplatin alone (Figure 10(b)). These results suggest that RE enhanced the efficacy of cisplatin and achieved greater CSC death than the drug alone.

There was no significant difference in the percentage of CSCs remaining between cells treated with cisplatin alone and cells treated with cisplatin combined with STE or between cells treated with cisplatin alone and cells treated with a combination of cisplatin and both extracts. This suggests that the greatest synergistic effect was observed when adding RE to cisplatin while STE and STE + RE had no synergistic effect. Importantly, this suggests that neither RE, STE, or both extracts combined interfered with the chemotherapeutic treatment from achieving CSC death.

The percentage of CD44-positive cells was significantly lower in cells treated with Taxol, a combination of STE and Taxol, a combination of RE and Taxol, and a combination of both extracts and Taxol than the untreated control. Most importantly, there was a significantly lower percentage of CSCs remaining after treatment with STE and Taxol combined than cells treated with Taxol alone. This suggests that STE positively interacted with Taxol, achieving significantly higher levels of CSC's death than the drug administered alone. All treatments except RE alone significantly reduced the percentage of CSCs compared to untreated cells, suggesting that CSCs are sensitive to STE alone as well as to a combination therapy with both extracts and common chemotherapeutics Taxol and cisplatin. Multiple studies report that CSCs can repopulate tumour masses following treatment with chemotherapeutics such as cisplatin and exhibit chemotherapeutic resistance [10, 14, 15]. Previous studies also suggested that differential survival of CSCs following chemotherapeutic treatment can increase the percentage of CSCs in progeny [14, 15]. Since these NHPs are well-tolerated and can be administered long-term, our results suggest that STE alone or in combination with RE can be administered post chemotherapeutic treatment to target CSCs in patients, thus reducing the chance of tumour mass repopulation and cancer relapse. These findings have implications for improving TNBC patient's prognosis because the observed synergistic herb-drug effects could allow for lower doses of a chemotherapeutic to be administered. Smaller doses of chemotherapeutics would decrease the onset of adverse side effects such as nausea and vomiting and would lower the risk of secondary infections that risk being lethal [47, 48]. Thus, a treatment comprised of either herb-drug combination has the potential to be well-tolerated, less toxic, and more effective at killing CSCs than current treatments, making it a strong candidate for long-term use in TNBC patients. Future studies should investigate whether CSCs develop resistance to treatment with RE and cisplatin combined or treatment with STE and Taxol combined. Future studies should also evaluate whether administering an herb-drug cocktail may be effective in avoiding the development of chemotherapeutic resistance, which would have further implications for reducing the risk of tumour relapse and treatment resistance.

In conclusion, both extracts, STE and RE induced apoptosis in MDA-MB-231 cells in a dose- and time-dependent manner. STE interacted positively with conventional chemotherapeutics Taxol and cisplatin, while RE did not interact with them at all. Therefore, combining STE with chemotherapeutics will result in greater anticancer effects. As well, this combination will allow for reduced chemotherapeutic dosages, thereby lowering the risk of toxic side effects. Most importantly, the combination of STE and RE displayed synergistic effects in terms of reducing cell viability in breast cancer. The STE-RE group was just as effective as the STE-RE-chemo group, thus removing the need for the drugs. With regard to their mechanisms of action, we found that STE induced apoptosis by targeting the MMP, while RE acted by inducing the production of ROS. Furthermore, cancer stem cells were also found to be sensitive to treatment with STE and a STE-RE combination. Notably, CSCs were more sensitive to treatment with a RE-cisplatin combination compared to cisplatin alone, as well as a STE-Taxol combination compared to Taxol alone. These potential synergistic effects against CSCs have implications for reducing the rate of tumour relapse and treatment resistance in TNBC patients and should be further explored in future studies.

Our results suggest that STE and RE can provide breast cancer patients with a sustainable and effective therapeutic regimen. Importantly, they may deal with the hurdles associated with chemotherapeutics, mainly, adverse effects negatively impacting a patient's health. The development of STE and RE as anticancer therapies may serve to improve the quality of life and long-term healthcare outcomes of breast cancer patients.

## Data Availability

The data used to support the findings of the study are available upon request.

## Supplementary Materials

Supplementary Materials HPLC analysis profiles and certificate of analysis (COA) for both extracts have been provided in Supplementary files.

## References

[1] Nguyen C. V. (2019). Anticancer activity of natural health products (dandelion root, lemongrass, and Hibiscus extracts); A study of efficacy, interaction, and mechanism of action. https://scholar.uwindsor.ca/etd/7725.

[2] Chavez K. J., Garimella S. V., Lipkowitz S. (2011). Triple negative breast cancer cell lines: one tool in the search for better treatment of triple negative breast cancer. *Breast Disease*.

[3] American Cancer Society (2021). Triple-negative breast cancer: details, diagnosis, and signs. https://www.cancer.org.

[4] Shah R. (2014). Pathogenesis, prevention, diagnosis and treatment of breast cancer. *World Journal of Clinical Oncology*.

[5] Alieva M., van Rheenen J., Broekman M. L. D. (2018). Potential impact of invasive surgical procedures on primary tumor growth and metastasis. *Clinical & Experimental Metastasis*.

[6] Kaya V., Yildirim M., Yazici G., Gunduz S., Bozcuk H., Paydaş S. (2018). Effectiveness of platinum-based treatment for triple negative metastatic breast cancer: a meta-analysis. *Asian Pacific Journal of Cancer Prevention: Asian Pacific Journal of Cancer Prevention*.

[7] Schneeweiss A., Ruckhäberle E., Huober J. (2015). Chemotherapy for metastatic breast cancer–an anachronism in the era of personalised and targeted oncological therapy?. *Geburtshilfe und Frauenheilkunde*.

[8] Cancer Research Uk (2020). *How Chemotherapy Works*.

[9] Weaver B. A. (2014). How taxol/Paclitaxel kills cancer cells. *Molecular Biology of the Cell*.

[10] Marzagalli M., Fontana F., Raimondi M., Limonta P. (2021). Cancer stem cells—key players in tumor relapse. *Cancers*.

[11] Reya T., Morrison S. J., Clarke M. F., Weissman I. L. (2001). Stem cells, cancer, and cancer stem cells. *Nature*.

[12] Yu Z., Pestell T. G., Lisanti M. P., Pestell R. G. (2012). Cancer stem cells. *The International Journal of Biochemistry & Cell Biology*.

[13] Kim E., Davidson L. A., Zoh R. S. (2015). Homeostatic responses of colonic LGR5^+^ stem cells following acute in vivo exposure to a genotoxic carcinogen. *Carcinogenesis*.

[14] Nedeljković M., Damjanović A. (2019). Mechanisms of chemotherapy resistance in triple-negative breast cancer—how we can rise to the Challenge. *Cells*.

[15] Rich J. N. (2016). Cancer stem cells. *Medicine*.

[16] Batlle E., Clevers H. (2017). Cancer stem cells revisited. *Nature Medicine*.

[17] Ma F., Li H., Wang H. (2014). Enriched CD44+/CD24− population drives the aggressive phenotypes presented in triple-negative breast cancer (TNBC). *Cancer Letters*.

[18] Chakrabarty A., Chakraborty S., Bhattacharya R., Chowdhury G. (2021). Senescence-induced chemoresistance in triple negative breast cancer and evolution-based treatment strategies. *Frontiers in Oncology*.

[19] Stewart R. L., Updike K. L., Factor R. E. (2019). A multigene assay determines risk of recurrence in patients with triple-negative breast cancer. *Cancer Research*.

[20] Biddle A., Gammon L., Fazil B., Mackenzie I. C. (2013). CD44 staining of cancer stem-like cells is influenced by down-regulation of CD44 variant isoforms and up-regulation of the standard CD44 isoform in the population of cells that have undergone epithelial-to-mesenchymal transition. *PLoS One*.

[21] Fultang N., Chakraborty M., Peethambaran B. (2021). Regulation of cancer stem cells in triple negative breast cancer. *Cancer Drug Resistance*.

[22] Koh M. Z., Ho W. Y., Yeap S. K., Ali N. M., Boo L., Alitheen N. B. (2021). Regulation of cellular and cancer stem cell-related putative gene expression of parental and CD44+CD24− sorted MDA-MB-231 cells by cisplatin. *Pharmaceuticals*.

[23] Li W., Ma H., Zhang J., Zhu L., Wang C., Yang Y. (2017). Unraveling the roles of CD44/CD24 and Aldh1 as cancer stem cell markers in tumorigenesis and Metastasis. *Scientific Reports*.

[24] Sheridan C., Kishimoto H., Fuchs R. K. (2006). CD44+/CD24-breast cancer cells exhibit enhanced invasive properties: an early step necessary for metastasis. *Breast Cancer Research*.

[25] Al-Hajj M., Wicha M. S., Benito-Hernandez A., Morrison S. J., Clarke M. F. (2003). Prospective identification of tumorigenic breast cancer cells. *Proceedings of the National Academy of Sciences*.

[26] Thapa R., Wilson G. D. (2016). The importance of CD44 as a stem cell biomarker and therapeutic target in cancer. *Stem Cells International*.

[27] Canadian Cancer Society (2016). *Chemotherapy and Other Drug Therapies: A Guide for People with Cancer*.

[28] Yeldag G., Rice A., Del Río Hernández A. (2018). Chemoresistance and the self-maintaining tumor microenvironment. *Cancers*.

[29] Storz P. (2005). Reactive oxygen species in tumor progression. *Frontiers in Bioscience*.

[30] Pignanelli C., Ma D., Noel M. (2017). Selective targeting of cancer cells by oxidative vulnerabilities with novel curcumin analogs. *Scientific Reports*.

[31] Ovadje P., Ammar S., Guerrero J.-A., Arnason J. T., Pandey S. (2016). Dandelion root extract affects colorectal cancer proliferation and survival through the activation of multiple death signalling pathways. *Oncotarget*.

[32] Alfarouk K. O., Verduzco D., Rauch C. (2014). Glycolysis, tumor metabolism, cancer growth and dissemination. A new pH-based etiopathogenic perspective and therapeutic approach to an old cancer question. *Oncoscience*.

[33] Liberti M. V., Locasale J. W. (2016). The Warburg effect: how does it benefit cancer cells?. *Trends in Biochemical Sciences*.

[34] Chen Z., Lu W., Garcia-Prieto C., Huang P. (2007). The Warburg effect and its cancer therapeutic implications. *Journal of Bioenergetics and Biomembranes*.

[35] Pandey M. M., Rastogi S., Rawat A. K. (2013). Indian traditional ayurvedic system of medicine and nutritional supplementation. *Evidence-based Complementary and Alternative Medicine*.

[36] Wachtel-Galor S., Benzie I. F. F. (2011). Herbal medicine: an introduction to its history, usage, regulation, current trends, and research needs. *Herbal Medicine: Biomolecular and Clinical Aspects*.

[37] Sereshti H., Samadi S., Jalali-Heravi M. (2013). Determination of volatile components of green, black, oolong and white tea by optimized ultrasound-assisted extraction-dispersive liquid–liquid microextraction coupled with gas chromatography. *Journal of Chromatography A*.

[38] Santos R. A., Andrade E. D. S., Monteiro M. (2021). Green tea (Camellia sinensis) extract induces p53-mediated cytotoxicity and inhibits migration of breast cancer cells. *Foods*.

[39] Ravindranath M. H., Saravanan T. S., Monteclaro C. C. (2006). Epicatechins purified from green tea (camellia sinensis) differentially suppress growth of gender-dependent human cancer cell lines. *Evidence-based Complementary and Alternative Medicine*.

[40] Liu L., Liu B., Li J. (2018). Responses of different cancer cells to white tea aqueous extract. *Journal of Food Science*.

[41] Ribeiro-Santos R., Carvalho-Costa D., Cavaleiro C. (2015). A novel insight on an ancient aromatic plant: the rosemary (rosmarinus officinalis L.). *Trends in Food Science & Technology*.

[42] Yesil-Celiktas O., Sevimli C., Bedir E., Vardar-Sukan F. (2010). Inhibitory effects of rosemary extracts, carnosic acid and rosmarinic acid on the growth of various human cancer cell lines. *Plant Foods for Human Nutrition*.

[43] Brindisi M., Bouzidi C., Frattaruolo L. (2020). Chemical profile, antioxidant, anti-inflammatory, and anti-cancer effects of Italian *Salvia rosmarinus* spenn. Methanol leaves extracts. *Antioxidants*.

[44] Philion C., Ma D., Ruvinov I. (2017). *Cymbopogon citratus* and *Camellia sinensis* extracts selectively induce apoptosis in cancer cells and reduce growth of lymphoma xenografts in vivo. *Oncotarget*.

[45] Ma D., Gilbert T., Pignanelli C. (2018). Exploiting mitochondrial and oxidative vulnerabilities with a synthetic analog of pancratistatin in combination with piperlongumine for cancer therapy. *The FASEB Journal: Official Publication of the Federation of American Societies for Experimental Biology*.

[46] Enciso-Benavides J., Alfaro L., Castañeda-Altamirano C. (2021a). Biological characteristics of a sub-population of cancer stem cells from two triple-negative breast tumour cell lines. *Heliyon*.

[47] Elhadi M., Khaled A., Msherghi A. (2021). Infectious diseases as a cause of death among cancer patients: a trend analysis and population-based study of outcome in the United States based on the surveillance, epidemiology, and end results database. *Infectious Agents and Cancer*.

[48] Lindley C. M., Hirsch J. D., O’Neill C. V., Transau M. C., Gilbert C. S., Osterhaus J. T. (1992). Quality of life consequences of chemotherapy-induced emesis. *Quality of Life Research*.

