# Supplementary material for: Green Tea Leaves and Rosemary Extracts Selectively Induce Cell Death in Triple-Negative Breast Cancer Cells and Cancer Stem Cells and Enhance the Efficacy of Common Chemotherapeutics
Source: Evid Based Complement Alternat Med. 2024 Jan 25;2024:9458716. doi: 10.1155/2024/9458716 (PMC11458307; doi:10.1155/2024/9458716)
Supplement: Supplementary Materials — HPLC analysis profiles and certificate of analysis (COA) for both extracts have been provided in Supplementary files. [file 9458716.f1.zip › Green Tea Ext - HPLC Chromatograms.pdf]

Catechins Calculation

23-02-2020

421-1437 421A200473

Components	Area Of Sample	Wt Of Sample (gms)	Area Of Ref	Wt Of Std (gms)	Purity Of Std (%)	%Component
**EGC**	259218		220657		12.11	**13.69**
**CAFFEINE**	1522591		1732057		7.00	**5.92**
**CATECHIN**	43963		83800		1.40	**0.71**
**EC**	225561	**0.0555**	306178	**0.0534**	3.34	**2.37**
**EGCG**	1616970		5024671		40.75	**12.62**
**GCG**	75883		218021		1.76	**0.59**
**ECG**	446120		1759199		11.22	**2.74**

Polyphenols = 76.48 %

Catechins = 32.71

Status:Man

SYNTHITE INDUSTRIES PVT LTD KOLENCHERY

Sample Information — Prominence-3

Acquired by : ROMARIO
Sample Name : CATECHIN STD
Sample ID : CATECHINS
Injection Volume : 20 uL
Vial Position :1
Data Filename : CATECHIN STD_001.lcd
Method Filename : CATECHINS.lcm
Date Acquired : 02/23/2020 10:27:56 AM
Data Processed : 02/23/2020 12:47:04 PM

Chromatogram

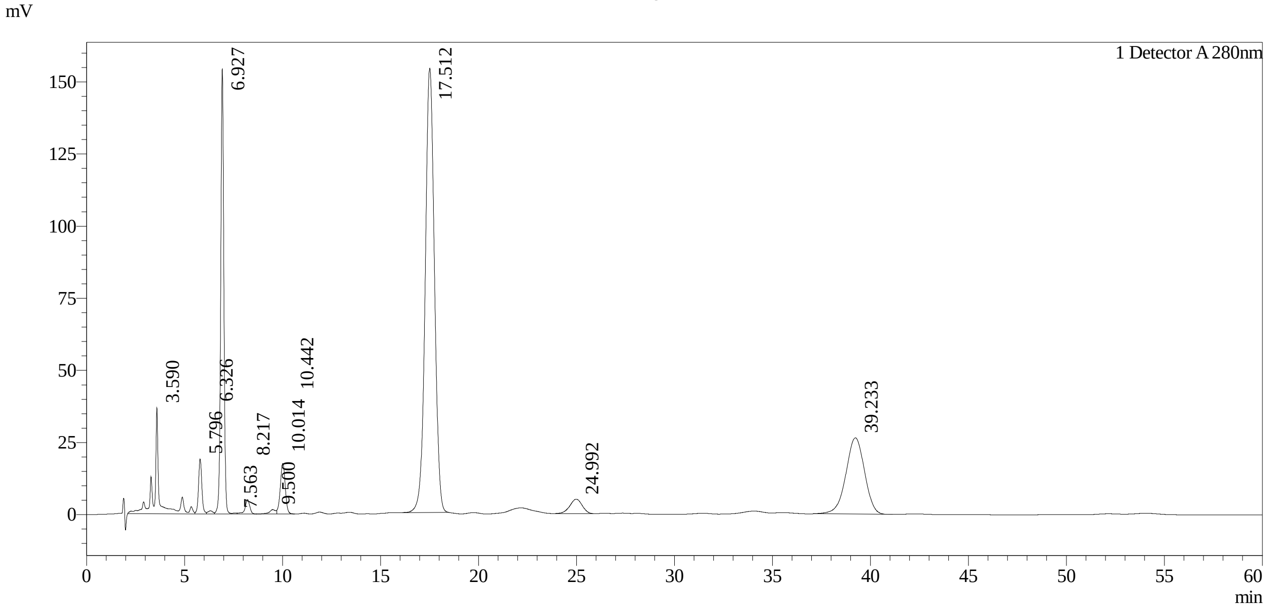

Peak Table

Detector A 280nm

Peak#	Name	Ret. Time	Area	Height	Conc.	Tailing Factor	Theoretical PlatES
1		3.590	668671	36703	6.638	1.100	5041
2		5.796	220657	19019	2.191	1.093	5922
3		6.326	18666	1064	0.185	--	2222
4		6.927	1732057	154406	17.196	1.012	8657
5		7.563	4901	332	0.049	--	453
6		8.217	83800	5048	0.832	--	6624
7		9.500	35251	1523	0.350	--	2911
8		10.014	306178	17558	3.040	--	7541
9		10.442	659	145	0.007	--	--
10		17.512	5024671	154010	49.884	1.018	6719
11		24.992	218021	4984	2.164	0.938	7305
12		39.233	1759199	26431	17.465	0.963	8111
Total			10072732	421222			

Status:Man

SYNTHITE INDUSTRIES PVT LTD KOLENCHERY

Sample Information

Prominence-3

Acquired by : ROMARIO
Sample Name : 421-1437 421k200473
Sample ID : CATECHINS
Injection Volume : 20 uL
Vial Position :2
Data Filename : 421-1437 421A200473_002.lcd
Method Filename : CATECHINS.lcm
Date Acquired : 02/23/2020 1:34:13 PM
Data Processed : 02/23/2020 2:50:55 PM

Chromatogram

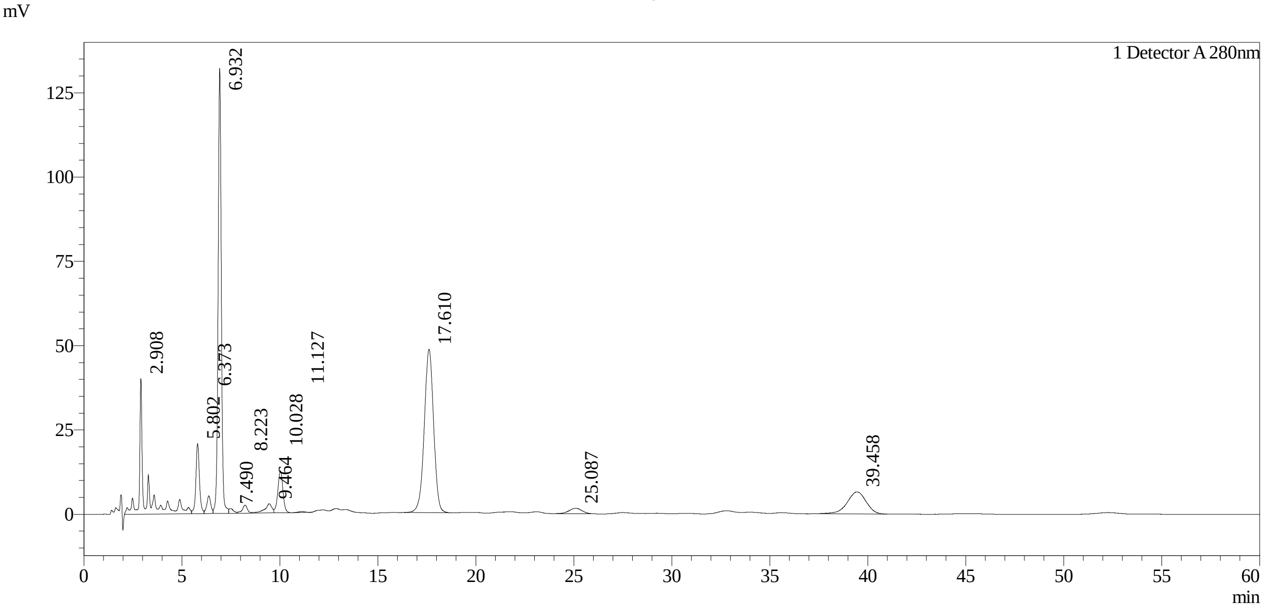

Peak Table

Detector A 280nm

Peak#	Name	Ret. Time	Area	Height	Conc.	Tailing Factor	Theoretical PlatES
1		2.908	698333	40226	13.795	1.235	3724
2		5.802	259218	20746	5.121	1.066	5406
3		6.373	75371	5283	1.489	--	4597
4		6.932	1522591	132039	30.077	1.016	8465
5		7.490	22098	1394	0.437	--	524
6		8.223	43963	2429	0.868	--	5812
7		9.464	70123	2742	1.385	--	4489
8		10.028	225561	12414	4.456	--	7150
9		11.127	6092	273	0.120	0.828	4351
10		17.610	1616970	48502	31.941	0.986	6582
11		25.087	75883	1641	1.499	0.996	6754
12		39.458	446120	6562	8.813	0.910	8082
Total			5062324	274251			
