# Supplementary material for: Green Tea Leaves and Rosemary Extracts Selectively Induce Cell Death in Triple-Negative Breast Cancer Cells and Cancer Stem Cells and Enhance the Efficacy of Common Chemotherapeutics
Source: Evid Based Complement Alternat Med. 2024 Jan 25;2024:9458716. doi: 10.1155/2024/9458716 (PMC11458307; doi:10.1155/2024/9458716)
Supplement: Supplementary Materials — HPLC analysis profiles and certificate of analysis (COA) for both extracts have been provided in Supplementary files. [file 9458716.f1.zip › Green tea Ext COA.pdf]

Synthite Industries Private Limited
Synthite Valley
Kolenchery, Cochin,Kerala,682311,India
T +91-484-2834200
F +91-484-2834351
E info@synthite.com
W www.synthite.com

Synthite

QUALITY ASSURANCE LABORATORY

SYN/QLC-024/E

Email : qchelpdesk@synthite.com

CERTIFICATE OF ANALYSIS

[This test report shall not be reproduced except in full without the written approval of the lab.The results in this report relate to the items tested]

**CUSTOMER**			
**MATERIAL**	Green Tea Matcha	**DESCRIPTION**	
		**INVOICE NUMBER**	
**QUANTITY**	0.100 KG	**TRADE NAME**	
**BATCH NUMBER**	421A200473	**CUST. MAT. CODE**	
**INSPECTION LOT NO**	40000092186	**BEST BEFORE**	
**MANUFACTURING DATE**		**PO REF No / DATE**	

Remarks

PHYSICAL AND ANALYTICAL DATA

SLNO	QUALITY PARAMETER	RESULT	TEST METHOD
	**Organoleptic Characteristics**		
1	Odour	Mild odour of tea	Sensory(QM/1011)
2	Colour-Visual	Greenish Yellow	General Inspection plan
3	Appearance	Fine powder	Sensory(QM/1011)
4	Taste	Bitter	Sensory(QM/1011)
	**Physical Characteristics**		
1	Tapped Bulk Density	0.7782 g/ml	ASTA 25.0, IVth Edition 1997
2	Loss On Drying	2.19 %	Moisture by gravimetry
	**Chemical Characteristics**		
1	Epi Galo Catechin Gallate	12.62 %	Food Research International
2	Total Catechins	32.71 %	Food Research International
3	pH of 1% Solution	5.40	pH Measurement
4	Caffeine	5.92 %	Liquid Chromatography
5	Polyphenols	76.48 %	Spectrometry
6	Granulometry (Sieving) Through 70 Mesh	98 %	ASTA Method No.10.0
7	Ash Content	<10 %	ASTA Method 3.0
8	Water Solubility At 1% Green Tea Matcha	>95 %	Visual Evaluation-Dispersibility
	**Microbiology**		

Approval Date : 23.02.2020

The result of batch no : 421A200473 corresponds to the sample submitted to the lab.

Prepared by :Mahesh

Microbiology by :Rachana

Approved by :Krishnakumar K R

Synthite Industries Private Limited Synthite Valley
Kolenchery, Cochin,Kerala,682311,India
T +91-484-2834200 E info@synthite.com
F +91-484-2834351 W www.synthite.com

QUALITY ASSURANCE LABORATORY

SYN/QLC-024/E

Email : qchelpdesk@synthite.com

CERTIFICATE OF ANALYSIS

[This test report shall not be reproduced except in full without the written approval of the lab.The results in this report relate to the items tested]

**CUSTOMER**			
**MATERIAL**	Green Tea Matcha	**DESCRIPTION**	
**QUANTITY**	0.100 KG	**INVOICE NUMBER**	
**BATCH NUMBER**	421A200473	**TRADE NAME**	
**INSPECTION LOT NO**	40000092186	**CUST. MAT. CODE**	
**MANUFACTURING DATE**		**BEST BEFORE**	
		**PO REF No / DATE**	

Remarks

PHYSICAL AND ANALYTICAL DATA

SLNO	QUALITY PARAMETER	RESULT	TEST METHOD
1	T. P. C	100 cfu/g	BAM,online January,2001 Chapter-3
2	T. Y. M. C	30 cfu/g	BAM, online, April, 2001 Chapter - 18
3	Enterobacteriaceae	<10 cfu/g	APHA IV th Edition
4	E.Coli	<3 mpn/g	BAM, online, February, 2013 Chapter - 4
5	Salmonella	Absent in 25g	BAM, online, August, 2016 Chapter - 5
6	Staphylococcus Aureus (Qualitative)	Absent in 25g	IS 5887 1976 (reaff 2005) PART II
	**Mycotoxin**		
1	Aflatoxin Total	<0.5 mg/kg	ASTA 24.2, 1997 Edition
	**Heavy Metals**		
1	Lead	<2 mg/kg	AOAC 19th Edition 999.11
2	Arsenic	<1 mg/kg	AOAC 19th Edition 999.11
3	Cadmium	<1 mg/kg	AOAC 19th Edition 999.11
4	Mercury	<0.1 mg/kg	AOAC 19th Edition 999.11

Approval Date : 23.02.2020

The result of batch no : 421A200473 corresponds to the sample submitted to the lab.

Prepared by :Mahesh   Microbiology by :Rachana   Approved by :Krishnakumar K R
