# Supplementary material for: Green Tea Leaves and Rosemary Extracts Selectively Induce Cell Death in Triple-Negative Breast Cancer Cells and Cancer Stem Cells and Enhance the Efficacy of Common Chemotherapeutics
Source: Evid Based Complement Alternat Med. 2024 Jan 25;2024:9458716. doi: 10.1155/2024/9458716 (PMC11458307; doi:10.1155/2024/9458716)
Supplement: Supplementary Materials — HPLC analysis profiles and certificate of analysis (COA) for both extracts have been provided in Supplementary files. [file 9458716.f1.zip › Rosemary Ext - HPLC Chromatogram.pdf]

==== Synthite Industies Pvt Ltd Analysis Report ====

D:\New folder\188 data\E\DATA\ROSMARINIC ACID\(08.01.20) Rosemary exrt.lcd

Acquired by	: Admin
Sample Name	: (08.01.20) Rosemary exrt
Sample ID	: Rosemary
Tray#	: 2
Vail #	: 52
Injection Volume	: 20 uL
Data File Name	: (08.01.20) Rosemary exrt.lcd
Method File Name	: ROSMARINIC ACID.lcm
Batch File Name	:
Report File Name	: Default.lcr
Data Acquired	: 1/8/2020 4:33:28 PM
Data Processed	: 1/8/2020 5:39:07 PM

<Chromatogram>

D:\New folder\188 data\E\DATA\ROSMARINIC ACID\(08.01.20) Rosemary exrt.lcd

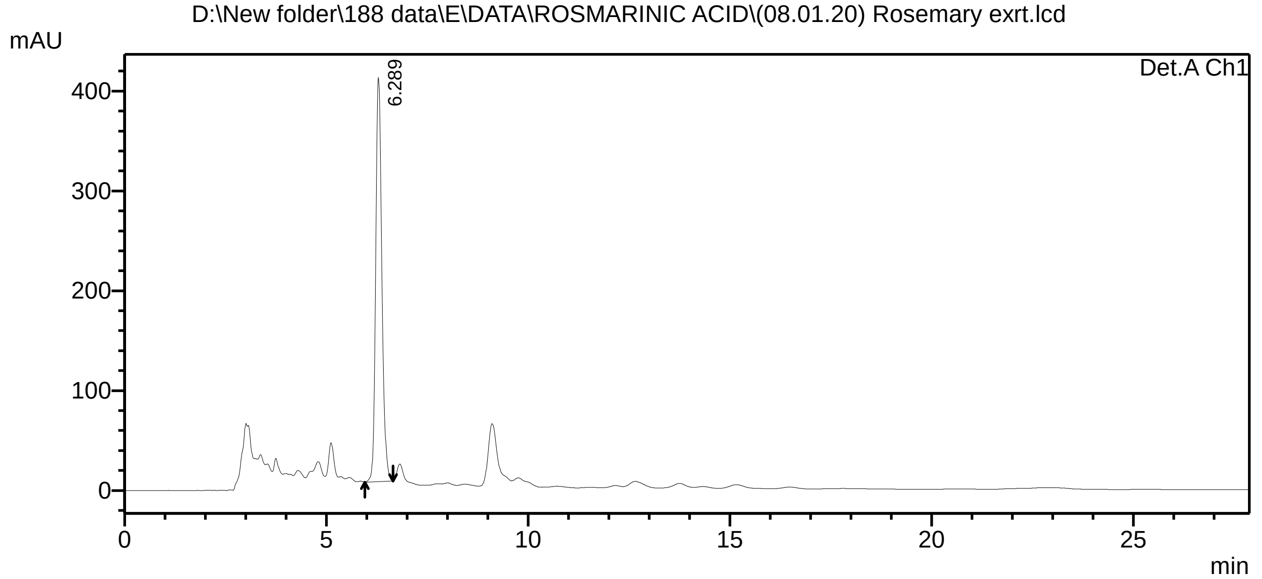

1 Det.A Ch1/330nm

PeakTable

Detector A Ch1 330nm

Peak#	Ret. Time	Area	Height	Area %	Height %
1	6.289	4267918	404796	100.000	100.000
Total		4267918	404796	100.000	100.000

%Rosmarinic acid = 4.6%
