# Supplementary material for: Green Tea Leaves and Rosemary Extracts Selectively Induce Cell Death in Triple-Negative Breast Cancer Cells and Cancer Stem Cells and Enhance the Efficacy of Common Chemotherapeutics
Source: Evid Based Complement Alternat Med. 2024 Jan 25;2024:9458716. doi: 10.1155/2024/9458716 (PMC11458307; doi:10.1155/2024/9458716)
Supplement: Supplementary Materials — HPLC analysis profiles and certificate of analysis (COA) for both extracts have been provided in Supplementary files. [file 9458716.f1.zip › Rosemary Ext COA.pdf]

Synthite Industries Private Limited
Marudur, Karamadai
Coimbatore,Tamil Nadu,641104,India
T +91 4254 276850
F +91 4254 276867
E info@synthite.com
W www.synthite.com

QUALITY ASSURANCE LABORATORY

SYN/QC 05(03)

Email : qchelpdesk@synthite.com

CERTIFICATE OF ANALYSIS

[This test report shall not be reproduced except in full without the written approval of the lab.The results in this report relate to the items tested]

**CUSTOMER**			
**MATERIAL**	Rosemary Ext	**DESCRIPTION**	
**QUANTITY**	0.100 KG	**INVOICE NUMBER**	
**BATCH NUMBER**	401H169472	**TRADE NAME**	
**INSPECTION LOT NO**	40000103298	**CUST. MAT. CODE**	
**MANUFACTURING DATE**		**BEST BEFORE**	
		**PO REF No / DATE**	

Remarks

PHYSICAL AND ANALYTICAL DATA

SLNO	QUALITY PARAMETER	RESULT	TEST METHOD
	**Organoleptic Characteristics**		
1	Appearance	Ok	Sensory(QM/1011)
	**Physical Characteristics**		
1	Brix	84.4 %	Spectrometry
2	Solubility	Soluble in water	Visual
	**Chemical Characteristics**		
1	Rosmarinic Acid	4.6 %	Liquid Chromatography
	**Residual Solvent**		
1	Residual Acetone	<20 mg/kg	FCC 12th Edition
	**Microbiology**		
1	T. P. C	<10 cfu/g	BAM,online January,2001 Chapter-3
2	T. Y. M. C	<10 cfu/g	BAM, online, April, 2001 Chapter - 18
3	E.Coli	<3 mpn/g	BAM, online, February, 2013 Chapter - 4
4	Salmonella	Absent in 25g	BAM, online, August, 2016 Chapter - 5
	**Mycotoxin**		
1	Aflatoxin-B1	<0.5 mg/kg	ASTA 24.2, 1997 Edition
2	Aflatoxin Total	<0.5 mg/kg	ASTA 24.2, 1997 Edition

Approval Date : 08.01.2020

The result of batch no : 401H169472 corresponds to the sample submitted to the lab.

P. Manojkumar

Prepared by :P. MANOJKUMAR

Microbiology by :DR K CHANDRASEKARAN

Approved by :SAJU K I

Synthite Industries Private Limited
Marudur, Karamadai
Coimbatore,Tamil Nadu,641104,India
T +91 4254 276850
F +91 4254 276867
E info@synthite.com
W www.synthite.com

QUALITY ASSURANCE LABORATORY

SYN/QC 05(03)

Email : qchelpdesk@synthite.com

CERTIFICATE OF ANALYSIS

[This test report shall not be reproduced except in full without the written approval of the lab.The results in this report relate to the items tested]

**CUSTOMER**			
**MATERIAL**	Rosemary Ext	**DESCRIPTION**	
**QUANTITY**	0.100 KG	**INVOICE NUMBER**	
**BATCH NUMBER**	401H169472	**TRADE NAME**	
**INSPECTION LOT NO**	40000103298	**CUST. MAT. CODE**	
**MANUFACTURING DATE**		**BEST BEFORE**	
		**PO REF No / DATE**	

Remarks

PHYSICAL AND ANALYTICAL DATA

SLNO	QUALITY PARAMETER	RESULT	TEST METHOD
	**Heavy Metals**		
1	Mercury	<1 mg/kg	AOAC 19th Edition 999.11
2	Cadmium	<1 mg/kg	AOAC 19th Edition 999.11
3	Arsenic	<1 mg/kg	AOAC 19th Edition 999.11
4	Lead	<2 mg/kg	AOAC 19th Edition 999.11

Approval Date : 08.01.2020

The result of batch no : 401H169472 corresponds to the sample submitted to the lab.

Prepared by :P. MANOJKUMAR

Microbiology by :DR K CHANDRASEKARAN

Approved by :SAJU K I
